# A Neurocomputational Model of the Effect of Cognitive Load on Freezing of Gait in Parkinson's Disease

**DOI:** 10.3389/fnhum.2016.00649

**Published:** 2017-01-09

**Authors:** Vignesh Muralidharan, Pragathi P. Balasubramani, V. Srinivasa Chakravarthy, Moran Gilat, Simon J. G. Lewis, Ahmed A. Moustafa

**Affiliations:** ^1^Department of Biotechnology, Indian Institute of TechnologyChennai, India; ^2^Parkinson's Disease Research Clinic, Brain and Mind Research Institute, University of SydneySydney, NSW, Australia; ^3^MARCS Institute for Brain and Behaviour and School of Social Sciences and Psychology, Western Sydney UniversitySydney, NSW, Australia

**Keywords:** Parkinson's disease, freezing of gait, basal ganglia, cognitive, motor, utility, conflict, cognitive load

## Abstract

Experimental data show that perceptual cues can either exacerbate or ameliorate freezing of gait (FOG) in Parkinson's Disease (PD). For example, simple visual stimuli like stripes on the floor can alleviate freezing whereas complex stimuli like narrow doorways can trigger it. We present a computational model of the cognitive and motor cortico-basal ganglia loops that explains the effects of sensory and cognitive processes on FOG. The model simulates strong causative factors of FOG including decision conflict (a disagreement of various sensory stimuli in their association with a response) and cognitive load (complexity of coupling a stimulus with downstream mechanisms that control gait execution). Specifically, the model simulates gait of PD patients (freezers and non-freezers) as they navigate a series of doorways while simultaneously responding to several Stroop word cues in a virtual reality setup. The model is based on an actor-critic architecture of Reinforcement Learning involving Utility-based decision making, where Utility is a weighted sum of Value and Risk functions. The model accounts for the following experimental data: (a) the increased foot-step latency seen in relation to high conflict cues, (b) the high number of motor arrests seen in PD freezers when faced with a complex cue compared to the simple cue, and (c) the effect of dopamine medication on these motor arrests. The freezing behavior arises as a result of addition of task parameters (doorways and cues) and not due to inherent differences in the subject group. The model predicts a differential role of risk sensitivity in PD freezers and non-freezers in the cognitive and motor loops. Additionally this first-of-its-kind model provides a plausible framework for understanding the influence of cognition on automatic motor actions in controls and Parkinson's Disease.

## Introduction

Deterioration of gait in Parkinson's Disease (PD) is of major concern as it severely affects the quality of life of patients. Some characteristics of gait disturbance include increased double support time, reduced stride length and velocity (Hausdorff et al., 1998; Morris et al., 1998). In addition to these features, freezing of gait (FOG) is a paroxysmal phenomenon where patients feel they are glued to the ground despite the desire to walk (Giladi et al., 2001; Nutt et al., 2011). A diverse range of environmental contexts can trigger FOG such as passing through narrow and confined spaces like doorways (Almeida and Lebold, 2010; Cowie et al., 2010; Shine et al., 2013a), turning and increased cognitive processing such as dual tasking (Schaafsma et al., 2003; Spildooren et al., 2010). The specific contribution of set-shifting, attention, visuo-spatial processing, sensory integration and emotions like anxiety have also been found to trigger FOG in PD (Lewis and Barker, 2009; Nutt et al., 2011; Martens et al., 2014).

Multiple neural networks are involved in gait processing and freezing including the sensory and motor cortices along with the association and prefrontal cortices, anterior cingulate cortex, basal ganglia and the brainstem (Shine et al., 2013b). Gait impairment in PD indicates a role for basal ganglia (BG) in these processes (Hausdorff et al., 1998; Morris et al., 1998). The cortico-basal ganglia system is organized as parallel loops associated with specific functional domains (Alexander et al., 1986; Parent and Hazrati, 1995; Graybiel, 1998). Competitive interactions among the cortico-basal-ganglia loops are thought to be a major factor for triggering freezing (Lewis and Barker, 2009). This is due to the impaired balance in demands to resources, that is, increased demands from cognitive, motor and limbic loops under depleted dopamine resources leading to the inhibition of brainstem locomotor systems (Lewis and Barker, 2009; Shine et al., 2013d). Several studies have investigated and reported deficits related to the effect of cognitive processes on gait in PD subjects especially freezers. The studies investigate the ability of these subjects to resolve conflict in the cognitive aspect of the task or in its interaction with a motor activity. Some examples include tasks such as the attention network task (ANT), the virtual reality gait task (Shine et al., 2013a), “timed up and go” task (TUG) (Weiss et al., 2010; Herman et al., 2011), object avoidance (Snijders et al., 2010; Pieruccini-Faria et al., 2014), and dual tasking (Yogev et al., 2005; Springer et al., 2006).

Our previous work (Muralidharan et al., 2013) on modeling PD gait explored the possibility of neuromodulator deficiency in PD freezers, particularly serotonin and norepinephrine. The model could explain deceleration and gait (step/stride length) changes observed in experiments involving walking through doorways with variable widths (Almeida and Lebold, 2010; Cowie et al., 2010). However, the role of cognitive factors was not included in our previous gait model. Furthermore, the prior model was only a Value based model, and did not consider the possible contributions of Risk (expected uncertainty), a quantity representing ambiguity associated with decision-making and is significant for understanding PD (Balasubramani et al., 2015). The current paper models gait performance in healthy controls and PD patients using Utility-based decision making that combines Value and Risk estimates for generating the decision variable toward executing behavior. Risk (or expected uncertainty) estimates capture the variance in reward outcome observed due to decision making (Balasubramani et al., 2014). Moreover, the sensitivity of the subjects to risk has previously been hypothesized to be a correlate of serotonin (5HT) function in the striatum. Based on the hypothesis that striatal serotonin levels code for risk-sensitivity, computational models have been able to unify several existing theories of serotonin function into a single theory (Balasubramani et al., 2014, 2015). In the experimental tasks modeled here, a word cue is associated with a specific motor action (walk/stop) resulting in a specific outcome (reward/punishment). Increased inconsistency in the relationship between cue and actions, results in greater reward variance or greater risk. Our model is built on the lines that this uncertainty in outcomes during the presentation of complex cue (also reflected as cognitive load as it demands the recruitment of more cognitive resources to achieve optimal behavior) facilitates the generation of a risk estimate. Along with the expected reward (or value) estimates, risk combines to generate the utility measure of decision process. The objective of the study is then to understand the interaction between cognitive and motor aspects in gait control, and analyze roles of conflict and load in the task design on simulated subjects (agent) as they approach a doorway. We also want to see whether the risk sensitivity of the subjects has any role in explaining the freezing behavior of certain PD patients and the implications of these measures on the therapeutic strategies. The results of the simulation are compared to behavioral results from PD patients (Matar et al., 2013; Shine et al., 2013d).

## Methods

### Computational modeling of the virtual reality gait task setup

The model simulates performance in a series of virtual reality experiments conducted on controls, PD non-freezers, and PD freezers (Matar et al., 2013; Shine et al., 2013d) to study the effects of conflict and cognitive load in PD patients. These behavioral experiments used a modified version of the Stroop task (Treisman and Fearnley, 1969) where there is an association of a color-word stimulus to a specific motor action (i.e., to walk or to stop) while subjects navigate a series of doorways. These virtual reality (VR) tasks assessed gait performance of the subjects, which require effective interaction between the cortico-basal ganglia circuits. The task setup for the model is inspired by two experiments (Matar et al., 2013; Shine et al., 2013d).

#### Patient description

In the Matar et al. (2013) experiement there were 18 healthy controls, 37 PD patients classified as non-freezers and 36 PD patients classified as freezers. The freezers were identified reliably using the item 3 of the FOG questionnaire (FOG-Q3—“Do you feel as if your feet are glued to the floor while walking, making a turn or while trying to initiate walking?”). Patients also performed the Mini mental State Examination (MMSE) and none had dementia according to the Movement Disorders Society PD dementia criteria.

The Shine et al. (2013d) experiment considered only PD non-freezers (*n* = 10) and freezers (*n* = 10) as part of the study. Since the study took place in both the ON and OFF conditions, clinically defined OFF condition was a minimum of 18 h without dopaminergic medication, with an average of 22.5 ± 3.1 h. Apart from the FOG questionnaire, the PD patients were also required to perform a few timed up and go trials with 180° left and right turns to identify patients with freezing behavior.

#### Model setup

**Figure 1:**
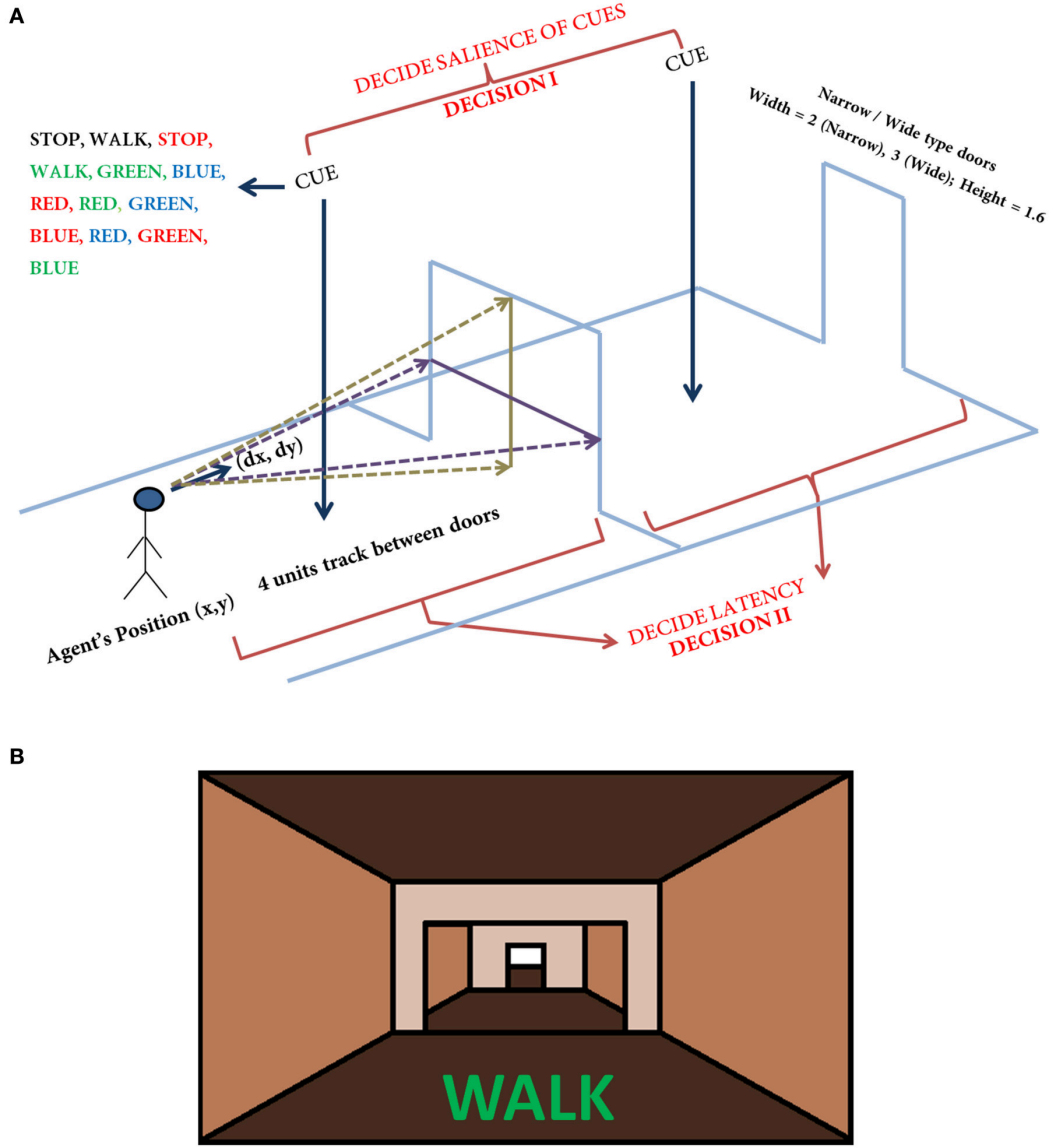
**The task setup (A)** used in the VR paradigm. The agent navigates a series of doorways (wide or narrow), while making decisions upon visualization of a cue or deciding the latency on traveling toward the doorway. The agent is presented with any one of the cue listed in the figure at random. The current position and the orientation of the agent are (X, Y) and (ΔX, ΔY) respectively. The doorways appearing could be narrow (2 units) or wide (3 units) again in a random fashion. The height of the doorways is kept constant at 1.6 units. A schematic of the view of doorways and cues **(B)** seen in the virtual reality by the subjects performing the task (adapted from Shine et al., 2013a).

In the simulation, the track that the agent navigates consists of 300 doorways (each doorway appearing at a distance of 4 length units). The agent (simulated subject; circular with about 1 unit in diameter) navigates a series of doorways (wide—3 length units and narrow—2 length units), while simultaneously performing the cognitive task of responding to word cues (Figure 1). At any time step, the output exhibited by the agent includes performing a forward motion (dubbed as a “step”), associated with a specific latency estimated as the number of time steps required to reach a decision threshold. The experiments gave the subjects, who were seated in front of a computer monitor, a first person view of the virtual reality (VR) environment which they could interact using a set of foot pedals. Alternate pressing of the foot pedals leads to forward motion in the VR setting, simulating the experience of locomotion, while the word cues were presented at the bottom of the monitor (Figure 1B). The model simulates this by executing a forward motion in the virtual environment. Additionally, to indicate walking or stopping within a trial, the task utilizes a set of simple and complex cues. The simple cues include the word “WALK” (usually presented in green) that indicated the subject to walk and the word “STOP” (usually presented in red) that indicated stopping. Simple cues are also presented in neutral color (e.g., BLACK). The task complexity is increased by interlacing simple cues with blocks of Stroop's words (complex cues). These words could be congruent (word and color are the same) or incongruent (word and color are different). Here, we use words RED, GREEN, and BLUE and the colors red, green and blue with their combinations providing a set of 13 different cues (see Figure 1 and Table 1). To represent a word stimulus, we adopt the following format “WORD (color).” The metric used for assessing freezing is inter-step length latency, defined as the time period between two consecutive alternating (left-right-left) presses of the foot pedals. Using this measure the following gait parameters are defined (Matar et al., 2013).

**Table 1:** **The list of cues used in the virtual reality paradigm and the actions associated with their appearance as used in the experiments and the model**.

**Type**	**Cues**	**Actions**	**References**
Simple	WALK, STOP, WALK, STOP	Direct associations	
Complex	Congruent: RED, GREEN, BLUE	Walk Walk/Stop	Matar et al., 2013 Shine et al., 2013d^*^
	Incongruent:RED, GREEN, BLUE, RED, GREEN, BLUE	Stop Stop/Walk	Matar et al., 2013 Shine et al., 2013d^*^

* *The blocks are counterbalanced so that half of the patients associate a congruent cue to walk and incongruent to stop and vice versa for the other half*.

- Modal Latency (preferred step latency): the mode of the latency distribution. It is assumed to be the baseline with respect to which a motor arrest or freeze episode is defined.
- Motor Arrest: an instance where the step latency is two times more than that of the modal latency (Shine et al., 2013d). This measure has shown good correlation to the amount of real freezing of gait in the classic “timed up and go” (TUG) tasks (Shine et al., 2013a).
- Maximum footstep Latency (MFSL): maximum latency exhibited within one to three steps following the presentation of a cue scaled to the modal footstep latency (Matar et al., 2013).

### Model architecture

**Figure 2:**
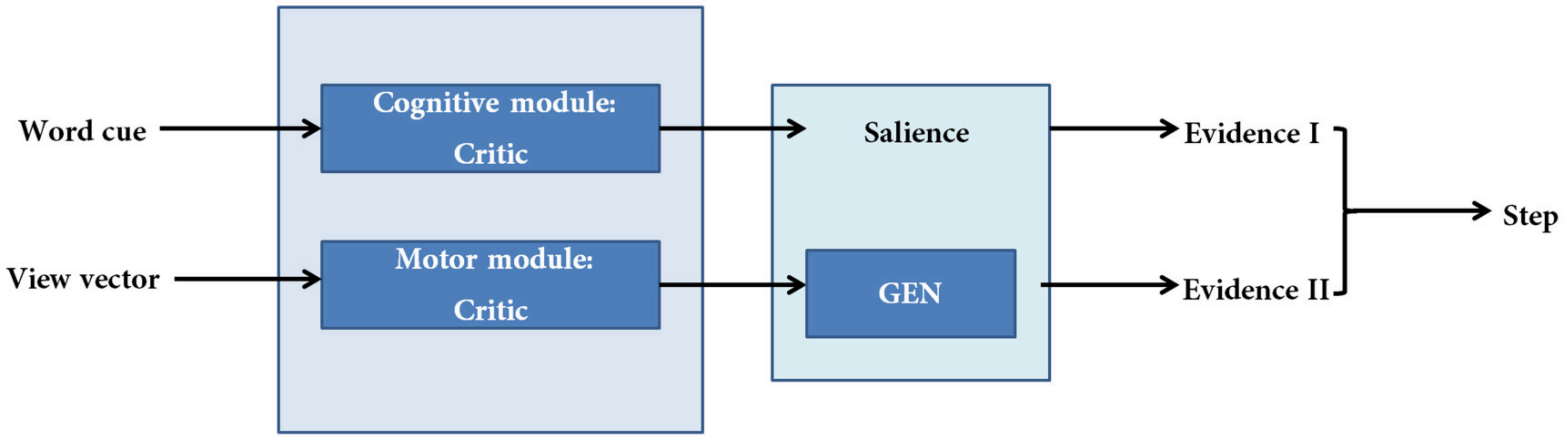
**A schematic of the model**. There are two modules (cognitive and motor) each making their respective evidences. The two evidences are combined subsequently to compute step latency.

The proposed cortico-basal ganglia model simulates the interaction between the motor and cognitive loops (using a “Motor Module” and “Cognitive Module”). Both the Cognitive and Motor Modules of the proposed BG model are based on the Actor-Critic architecture, each having its respective Critic and Actor. Evidences from the two modules are combined to execute the final output. These two modules build their respective evidences based on different sensory stimuli—the Motor Module based on visual appearance of the doorway, and the Cognitive Module based on the word cue. The first evidence (EI) involves the Cognitive Module identifying the salience of a word cue upon its presentation (Figure 2). Since a word cue does not appear at every moment, EI is taken only upon the presentation of the word cue. The second evidence (EII) involving the Motor Module takes the visual appearance of the doorway as input and computes the direction of the step as well as the latency associated with it as outputs (Figure 2). EII is computed at every time step. The GEN (Go/Explore/Nogo) policy, which is the Actor, adopts hill-climbing over the Utility landscape to calculate the velocity of the agent. The evidences of the two modules are combined subsequently to get the step latency.

Below we describe the following modeling components: (1) Utility-based decision making, (2) Cognitive Module, (3) Motor Module, (4) computations of gait parameters, and (5) Modeling PD condition.

## Utility-based decision making

The Value function “*Q*” under a policy π, which is defined as the expected discounted sum of rewards, associated with a state, “*s*,” and an action, “a,” pair, at time, “*t*” is given as,

(1) $$\begin{array}{l} Q^{\pi }(s,a)=E_{\pi }(r(t+1)+\gamma \;r(t+2)+\gamma^{2}\;r(t+3)\\ \;+...|s(t)=s,a(t)=a) \end{array}$$

where, *r* is a scalar reward obtained at time *t*, γ, is the *discount factor* controlling the time scale of reward prediction (Sutton and Barto, 1998). The update form of the Value function is as follows:

(2) $$\begin{array}{r} Q\left(t+1\right)=Q\left(t\right)+\eta_{Q}\delta \left(t\right) \end{array}$$

where, η_*Q*_ is the learning rate and 'δ' is the temporal difference (TD) error.

(3) $$\begin{array}{r} \delta \left(t\right)=r\left(t\right)+\gamma Q\left(t+1\right)-Q\left(t\right) \end{array}$$

or

(4) $$\begin{array}{r} \delta \left(t\right)=r\left(t\right)-Q\left(t\right) \end{array}$$

for instantaneous rewards.

The Utility function formulation combines the Value function defined above with the Risk function, *h*, which represents reward variance (Bell, 1995; d'Acremont et al., 2009). In recent work, we showed that the Utility function formulation can be effectively used to model the interactions between dopamine and serotonin in BG (Balasubramani et al., 2014, 2015).

The Risk function is updated as follows:

(5) $$\begin{array}{r} h\left(t+1\right)=h\left(t\right)+\eta_{h}\xi \left(t\right) \end{array}$$

where, ξ(*t*) is the Risk prediction error given by:

(6) $$\begin{array}{r} \xi \left(t\right)=\delta {\left(t\right)}^{2}-h\left(t\right) \end{array}$$

We proposed a slightly modified form of Utility “*U*,” expressed as a combination of the Value function and the Risk function weighted again by the function sign(*Q*), as follows:

(7) U(t)=Q(t)-α sign(Q(t))h(t)

where, α that controls the risk sensitivity representing the functioning of serotonin (5HT) in the BG (Balasubramani et al., 2014). The *sign*() term in Equation (7) represents the non-linear risk sensitivity (Balasubramani et al., 2014).

## The cognitive module

**Figure 3:**
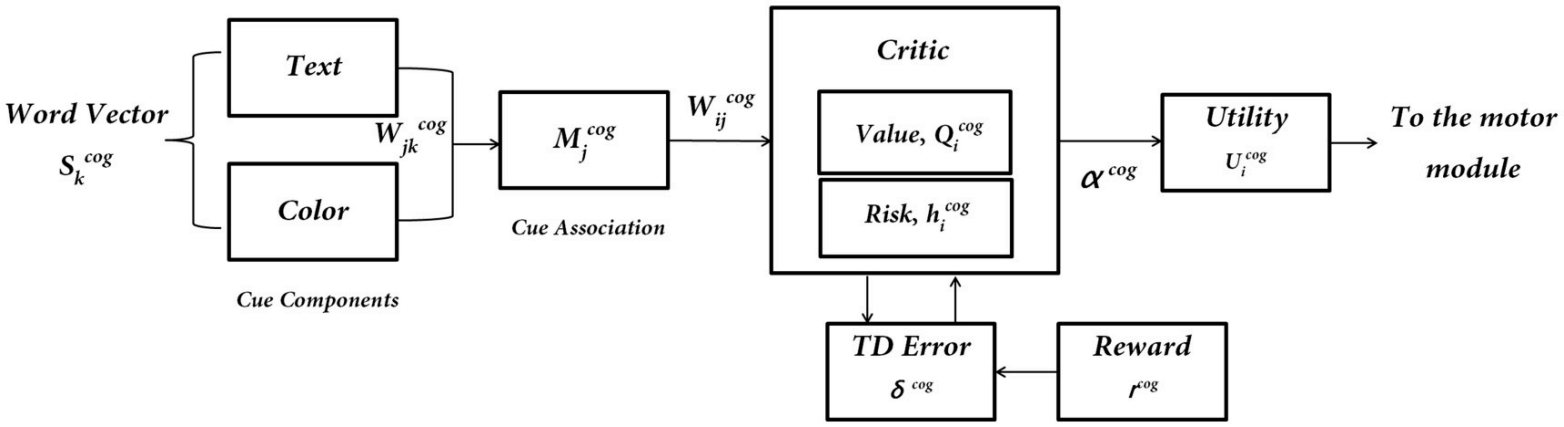
**The subcomponents of the Cognitive Module: input is the word cue (text + color)—$S_{k}^{cog}$; “Critic Module” computes Action Values and Risk; “Utility Module” combines Action Values ($Q_{i}^{cog}$) and Risk ($h_{i}^{cog}$) to compute Utility ($U_{i}^{cog}$), which is the output of the Cognitive Module**.

The Cognitive Module is a two-layer neural network (Figure 3) that receives its input as the word cues, which is a color-word pair defined in the format WORD (color). It returns as output the Utility associated with the action “walk.” The word stimulus is represented as a 9-dimensional vector $S_{k}^{cog}$). The first 5 bits correspond to the word inputs (STOP, WALK, RED, GREEN, and BLUE) and the last 4 bits correspond to the color associated with that word (red, green, blue and neutral). The respective bits are turned to 1's for a specific color-word stimulus. The input is fed into an association layer ($M_{j}^{cog}$) which consists of 5 nodes, with sigmoidal non-linearity. The output ($Q_{i}^{cog}$) consists of 2 nodes representing the Action Values for walking and stopping, *i* ε [w, s]. In accordance to the experiments, upon visualization of a cue, the agent has two actions to select from, that is, to walk or to stop.

### Critic

The simulated agents performing the VR experiments are trained to associate the cues with actions to at least an accuracy level of 95%. The network gives the Action Values for the cue that is *Q*_*w*_ and *Q*_*s*_. The weights ($W_{jk}^{cog}$ and $W_{ij}^{cog}$) of the network are randomly initialized and upon presentation of a cue ($S_{k}^{cog}$), one of the output nodes ($Q_{i}^{cog}$) is selected via competitive dynamics. Here *S*^*cog*^ represents the input word vector, and *M*^*cog*^ denotes the hidden layer (association layer) of the neural network.

(8) $$\begin{array}{r} M_{j}^{cog}\left(t\right)=g\left(W_{jk}^{cog}S_{k}^{cog}\left(t\right)\right) \end{array}$$

(9) $$\begin{array}{r} Q_{i}^{cog}\left(t\right)=A_{Q}^{cog}g\left(W_{ij}^{cog}M_{j}^{cog}\left(t\right)\right) \end{array}$$

where $g\left(x\right)=\frac{1}{1+e^{-\lambda^{cog}x}}$ and λ^*cog*^ is the slope of the sigmoid function. The Qs are initially trained by selecting a node through the forced alternative choice method. A reward (*r*^*cog*^) is obtained upon selecting an action (*r*^*cog*^ = 1 for the correct action; *r*^*cog*^ = 0 for the incorrect action). The weights for the corresponding node are updated with learning rate η^*cog*^ using the following rule:

(10) $$\begin{array}{r} \Delta W_{ij}^{cog}=\eta^{cog}\delta_{i}^{cog}\left(t\right)M_{j}^{cog} \end{array}$$

(11) $$\begin{array}{r} \Delta W_{jk}^{cog}=\eta^{cog}\delta_{j}^{cog_{2}}\left(t\right)S_{k}^{cog} \end{array}$$

where the prediction error defined as, $\delta^{cog}\left(t\right)=r^{cog}\left(t\right)-Q_{i}^{cog}\left(t\right)$, is used to update the output weights ($W_{ij}^{cog}$) and is backpropagated as δjcog2(t)=Σ iWijcogg′(WjkcogSkcog(t))δis(t) for updating the weights $W_{jk}^{cog}$. The prediction error δ^*cog*^ is an analog of temporal difference error correlated with dopamine signaling in the Cognitive Module (Schultz, 2010).

The training procedure is as follows:

1. The Cognitive Module is trained initially for approximately 600 trials on only the simple cues (Table 1) which include the WALK (neutral), STOP (neutral), WALK (green) and STOP (red) appearing in random order. This training biases the network toward the implicit responses toward a WALK or a STOP cue and the color in which it is presented.
2. The network is then subjected to the complex (congruent and incongruent) cues (Table 1) for additional 1000 trials. Further, in this step the simple and the congruent cues are presented more frequently (2:1) than the incongruent cues. This is done to ensure consistency with the behavioral experiments.
3. The selection of a node “*i*” at the output ($Q_{i}^{cog}$) leads to changes in the weights ($W_{ij}^{cog}$) for only that particular node at the output level; however, all the weights ($W_{jk}^{cog}$) from the input to the association layer are updated.

After learning the *Q*-Values, the network can in parallel be used to compute the Utility for the cues using the following approach. Since there is an uncertainty in the task toward the identification of the appropriate action for a Stroop word, this uncertainty is calculated by the probability of walking (*p*_*w*_) for a particular cue.

(12) $$\begin{array}{r} p_{w}=\frac{Q_{w}}{Q_{w}+Q_{s}} \end{array}$$

The Risk function (*h*^*cog*^) is then estimated by the following expression.

(13) $$\begin{array}{r} h^{cog}\left(t\right)=\frac{p_{w}\left(1-p_{w}\right)}{a_{p}} \end{array}$$

*a*_*p*_ is a constant used to scale up the Risk function so that the maximum Risk is 1. The parameter *a*_*p*_ is set to be 0.25 in all simulations. The Utility is then defined for the cognitive network as a combination of the Value (*Q*^*cog*^) and the uncertainty/Risk function (*h*^*cog*^). The amount of Risk taken into account for the Utility computation is controlled by the sensitivity factor α^*cog*^ similar to the manner done in (Balasubramani et al., 2014).

(14) $$\begin{array}{r} U^{cog}\left(t\right)=Q^{cog}\left(t\right)-\alpha^{cog}sign\left(Q^{cog}\left(t\right)\right)\sqrt{h^{cog}\left(t\right)} \end{array}$$

The Utility is used as a measure of the salience of a cue and is the EI of the task (Figure 2). It represents information regarding the decision to be taken toward a specific stimulus upon its presentation. The Utility for walking ($U_{w}^{cog}$) is further passed on to the motor network to estimate step latency (See Supplementary Material S2: Table S1 for the list of parameter values to simulate the Cognitive Module).

## The motor module

The ability of the agent to navigate through a series of doorways based on the visual appearance of the doorway is controlled by the Motor Module. The virtual reality paradigm used several different characteristics of the doorway such as wide and narrow doorways, wide and narrow passages and also sliding doorways which open upon approaching the doorway (Matar et al., 2013; Shine et al., 2013d). For simplicity, in the model we consider only two different types of doorways, a wide and a narrow one; sliding doorways are omitted. We assume that the only property of the doorway that determines freezing or non-freezing is its width.

The agent is associated at every point on the track with a heading direction/ velocity Δ*Z*(*t*) = [Δ*X*(*t*) Δ*Y*(*t*)] (where *X* is the dimension along the track in the forward direction and *Y* is perpendicular to it (Figure 1) which points to the direction in which the agent is moving (or looking) at the moment. Importantly, a successful passage through a doorway yields a reward (*r*^*mot*^ = 1) and collision with the sides leads to punishment (*r*^*mot*^ = −1). Using such a reward scheme, the agent constructs a Value function and navigates through the virtual corridor. So although in the experiments the subjects looked straight and experienced only forward motion, in the model the agent had a 2D motion. It was necessary for the virtual agent to successfully pass through the doorway which yielded a positive reward and bump with the sides that gave it negative reward or punishment, which gave rise to a speed-accuracy tradeoff close to the doorway resulting in the agent slowing naturally as it approaches the doorway. In previous studies, this approach helped explain deceleration of PD freezers as they approached a narrow doorway (Muralidharan et al., 2013).

**Figure 4:**
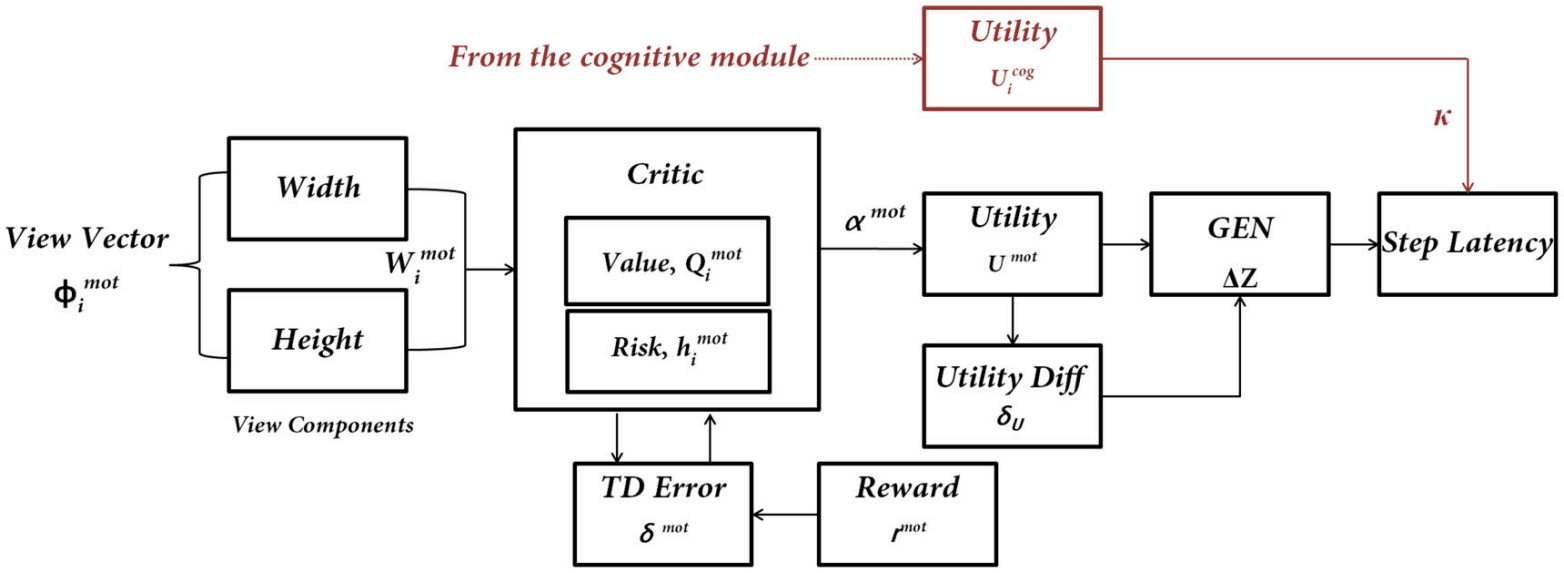
**The subcomponents of the Motor Module**. The input to the network is the “view vector” associated with the doorway. The Critic Module computes Action Value and Risk, and combines the two into Utility function. The next step of the agent [ΔZ = (ΔX, ΔY)] is computed by using a policy (GEN Module). The next step information is converted to Step Latency, which represents the evidence (EII) of the Motor Module. Utility from the Cognitive Module modulates the Step Latency whenever a word cue is presented.

Since the step latency is the desired parameter it is estimated near the doorway using the following approach. A region of around 0.1 length units on either side of a doorway is considered as a significant distance to isolate the effect of the doorway on reaction times. In this region, the maximum latency exhibited by the agent is averaged across trials for the two doorway types. In order to study the effect of cues on the latency near the doorways, the agent is made to navigate through the track while simultaneously presented with different cues. Within a single trial (from the start point until encountering a doorway) the cues always appear at a distance of about 2 length units before the doorway. Unlike the Cognitive Module where the action occurs at discrete steps when a word cue is presented, the actions of the Motor Module are made every time step, since the doorway is continuously visible to the agent (Figure 4).

### Cue-visual input

**Figure 5:**
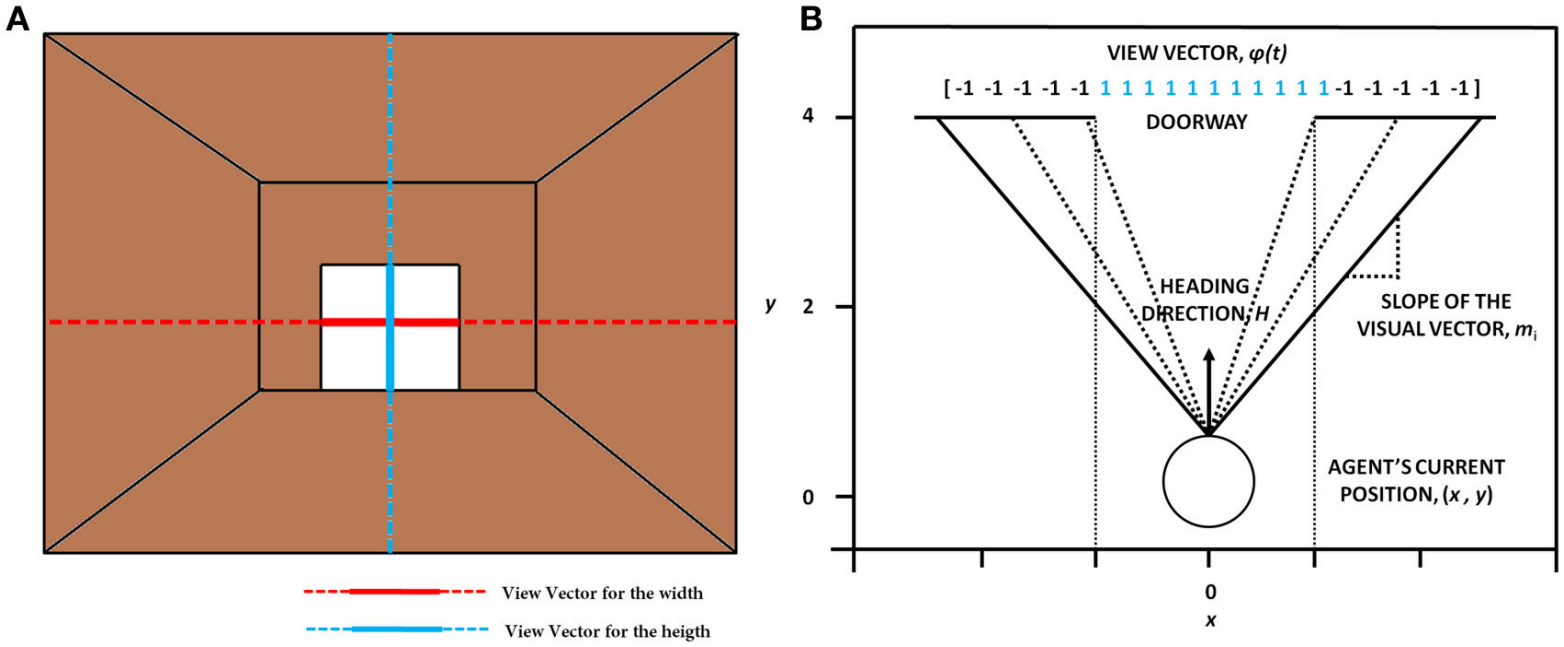
**The concept of visualizing the doorway (A)** which shows the orthogonal lines that depict the view in terms of width (in red) and height (in blue). **(B)** The construction of the view vector given a heading direction H, the agent's current position (x, y) and the width of the doorway (w^*door*^).

The *visual information represented by the “view vector,”* ϕ, acts as the state for the Critic of the Motor Module. The agent can see around 120° along the width and 90° along the height. Both the horizontal and vertical fields of vision are split into 50 sectors each. Thus, the visual vector is a 100-dimensional binary vector [ϕ ε (−1 1)], where the first 50 bits code the width of the doorway visualized by the agent and the last 50 code for the height of the doorway. The heading direction vector (*H*) determines the direction the agent is looking, at a particular time step. The corresponding bits in the view vector are switched on (=1) whenever a doorway is in the field of vision of the agent (Figure 5). The factor of height played a role in distinguishing the discrepancy in the code which might occur in certain conditions. Since the height of the doorways remained the same throughout the simulations, the view of a narrow doorway visualized close by can be differentiated from seeing a wide doorway from far away. The number and location of 1's in the visual field becomes a function of the position and the orientation of the agent and forms an implicit code for representing space. A conceptual illustration and the construction of the visual vector is seen in Figure 5. See Supplementary Material S1 for the construction of the view vector.

### Critic

The Critic in the Motor Module is slightly different from that of the Cognitive Module. The Critic of the Motor Module, unlike the Critic in the Cognitive Module, estimates both Value and Risk as a function of the view vector. This distinction is present since the views close to the doorways themselves are sufficient to encode the attribute of Risk because of high probability of hitting the sides on approaching a doorway. The Critic computes the Value “*Q*^*mot*^” for the view vector [ϕ(t)]. It is defined as an estimation of the predicted reward at any time, *t*, for that state ?(t). We model *Q*^*mot*^(t) as in Equation (15):

(15) $$Q^{mot}(t)=A_{Q}^{mot}f(\sum W_{l}^{value}(t)\phi_{l}(t))$$

where $f\left(x\right)=\frac{1}{1+e^{-\lambda^{mot}x}}$ and λ^*cog*^ is the slope of the sigmoid function. The update equation for the weights in the above approximation (having weight vector, *W*^*value*^) is given by Equation (16):

(16) $$\begin{array}{r} \Delta W^{value}=\eta^{mot}\delta^{mot}\phi \left(t\right) \end{array}$$

Here, η^*mot*^ is the learning rate for the critic and “δ^*mot*^” denotes the temporal difference (TD) error in Value function, that has been linked to dopamine signaling (Schultz, 2010). It is given by Equation (17) in which γ is the discount factor.

(17) $$\begin{array}{r} \delta^{mot}=r^{mot}\left(t\right)+\gamma Q^{mot}\left(t\right)-Q^{mot}\left(t-1\right) \end{array}$$

The Risk function for the Motor Module is approximated using the Equation 18. The weights for the Risk computation are updated using (δ^*mot*^)^2^ (Equation 19). Note that this same quantity is estimated as uncertainty in the Cognitive Module (Equation 13).

(18) $$h^{mot}(t)=A_{h}^{mot}f(\sum W_{i}^{risk}(t)\phi_{i}(t))$$

(19) $$\begin{array}{r} \Delta W^{risk}=\eta^{mot}{\left(\delta^{mot}\right)}^{2}\phi \left(t\right) \end{array}$$

The Utility function combines the Value function and α^*mot*^ controlled Risk function.

(20) $$\begin{array}{r} U^{mot}\left(t\right)=Q^{mot}\left(t\right)-\alpha^{mot}sign\left(Q^{mot}\left(t\right)\right)\sqrt{h^{mot}\left(t\right)} \end{array}$$

### Actor/go explore NoGo policy

The Actor in the model computes the direction of movement for the agent using the latency is estimated. We assume in the model that there must be an actor only for the Motor Module, and the Cognitive Module influences this information based on the word stimulus that appeared during the trial, thereby controlling the latency of the motor actions. The Actor dubbed as GEN or the GO/EXPLORE/NOGO policy is a type of action selection mechanism which performs a stochastic hill-climbing over the Value function space. This type of action selection has been shown to model a range of BG functions in healthy controls and PD patients (Sridharan et al., 2006; Magdoom et al., 2011; Kalva et al., 2012; Gupta et al., 2013; Muralidharan et al., 2013). In the present model, however, the GEN policy is used to maximize the Utility rather than the Value function, as in our prior models (Balasubramani et al., 2014, 2015). This is achieved by modifying the GEN equations of Muralidharan et al. (2013) and extending them to the Utility function as follows:

(21) $$\begin{array}{r} \delta_{U}=U^{mot}\left(t\right)-U^{mot}\left(t-1\right) \end{array}$$

The 3 regimes of action selection (GO/EXPLORE/NOGO) can be represented as a function of δ_*U*_ by the following expression

(22) $$\begin{array}{l} \Delta Z\left(t\right)=A_{G}sig\left(\lambda_{G}\delta_{U}\right)\;\Delta Z\left(t-1\right)\\ \;+A_{E}\chi \exp\left(-\delta_{U}^{2}/\sigma_{E}^{2}\right)\\ \;-A_{N}sig\left(\lambda_{N}\delta_{U}\right)\;\Delta Z\left(t-1\right) \end{array}$$

where *A*_*G*_, *A*_*N*_, *A*_*E*_ are the gains of GO, NOGO and EXPLORE regimes respectively, λ_*G*_ and λ_*N*_are the sensitivities of GO and NOGO regimes and σ_*E*_ is the parameter controlling the extent of exploration. Δ*Z*(*t*) = [Δ*X*(*t*) Δ*Y*(*t*)] represents the change in position at the time step *t* which includes both the components of velocity, using which the current position of the agent is updated. The GEN policy can give rise to negative velocities and thus can hamper the agent's movement by inducing backward motion in the simulations. In order to prevent the agent from doing this, the y component of the velocity [Δ*Y*(*t*)] is passed through a sigmoidal function before addition to the position [*Z*(*t*)]

(23) $$\begin{array}{r} \overset{\sim }{\Delta Y}\left(t\right)=\frac{1}{1+e^{\left(-\lambda^{vel}\Delta Y\left(t\right)\right)}} \end{array}$$

(24) $$\begin{array}{r} \Delta Z\left(t\right)=\left[\Delta X(t)\;\overset{\sim }{\Delta Y}\left(t\right)\right] \end{array}$$

(25) $$\begin{array}{r} Z\left(t+1\right)=Z\left(t\right)+\Delta Z\left(t\right) \end{array}$$

Here *Z* = (*X*,*Y*) and denotes the position of the agent on the track. This gives rise to a different orientation and a view vector and thus the cycle continues. The Δ*Z*(*t*) represents EII of the task (Figure 2) and is used to estimate the step latency. (See Supplementary Material S2: Table 1 for the list of parameter values to simulate the Motor Module)

## Estimating the latency of motor actions

The final output of the model is the step latency which is dependent on the outputs of both the Cognitive and Motor Modules (Figure 4). Since the words appear only at certain instants in the task, their contribution to the latency is maximal only at the time of their presentation. A decision variable, *s*, which can be thought to accumulate evidence for an action is used to get the reaction times from the model. From the Motor Module, the GEN output is used to estimate the latency at any given point in time as,

(26) $$\begin{array}{l} \overset{•}{s}=\kappa *\left\|\Delta Z(t)\right\| \end{array}$$

(27) $$\begin{array}{r} \kappa =U_{W}^{cog}+b \end{array}$$

In Equation (26), the variable “*s*” is defined as “intent for walking” (product of the Utility from the Cognitive Module and the GEN output from the Motor module) as its rate of change indicates how fast the agent would take a forward step, and has to cross a threshold (*th* = 1) for the action to be executed. The time taken for “*s*” to cross the threshold is the “step latency.” The velocity term Δ*Z*(*t*) comes from the Motor Module, and the coefficient “κ” comes from the Cognitive Module. Upon the appearance of a word, the $U_{W}^{cog}$ is produced by the Cognitive Module which is used along with the velocity to compute the latency. At instants when there is no word cue, κ is set to the default value of *b* (Equation 27). Thus, the walking latency is determined by contributions from both Motor and Cognitive Modules.

## Modeling PD

Parameters that represent PD conditions (freezers and non-freezers) in the model include the temporal difference errors in both motor (δ^*mot*^) and cognitive (δ^*cog*^) modules and sensitivity parameters for the Risk function in the Utility computation (α^*mot*^ and α^*cog*^). In agreement with previous modeling efforts, the temporal difference error is appropriately clamped to simulate dopamine deficient conditions (Gupta et al., 2013; Muralidharan et al., 2013; Balasubramani et al., 2014), using the factor δ^*^. Therefore, if [a, b] represents the range of dopamine levels in healthy controls then [a, δ^*^] represents the PD OFF condition, where δ^*^ < b. The PD ON condition is modeled by the addition of a medication factor δ_*med*_ to the existing dopamine level.

(28) $$\begin{array}{r} \text{PD OFF}:\begin{array}{c} If\;\delta >\delta^{*}\\ \delta =\delta^{*} \end{array} \end{array}$$

(29) $$\begin{array}{cc} \text{PD ON}: & \begin{array}{l} If\;\delta >\delta^{*}\\ \;\delta =\delta^{*}+\delta_{med}\\ else\\ \;\delta =\delta +\delta_{med} \end{array} \end{array}$$

In addition to these parameters, from our previous work on modeling PD freezers, the exploration factor σ_*E*_ (Equation 22) is also considered as a factor contributing to FOG behavior (Muralidharan et al., 2013).

**Figure 6:**
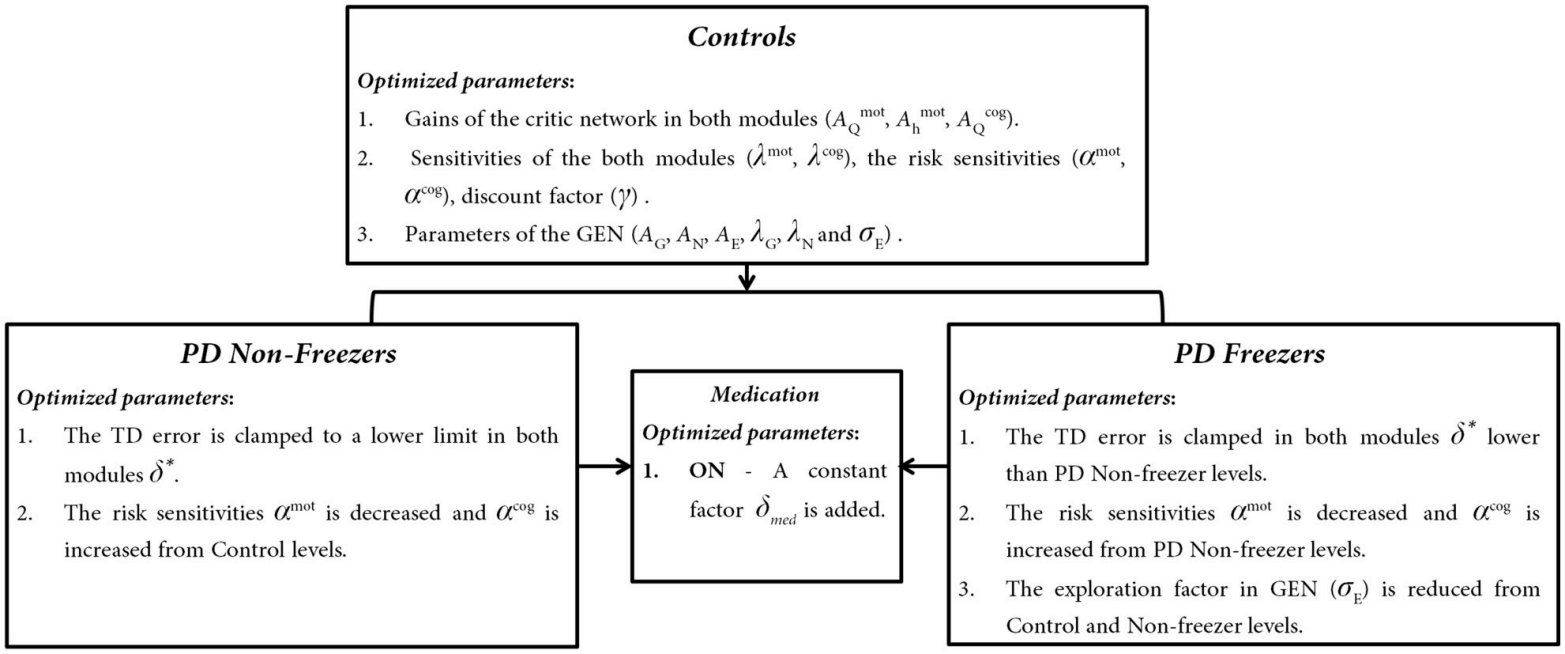
**The trends observed in optimized parameters involved in Controls, PD Non-freezer and PD freezer conditions**.

Model parameters representing the Motor and Cognitive modules in (1) healthy controls, (2) PD ON, and (3) PD OFF conditions, are estimated as follows. The critical parameters including the gains of the critic network in both modules ($A_{Q}^{mot}$, $A_{h}^{mot}$, $A_{Q}^{cog}$), the sensitivities of the critic networks (λ^*mot*^, λ^*cog*^), the risk sensitivities (α^*mot*^, α^*cog*^), discount factor (γ) and the parameters of the GEN (*A*_*G*_, *A*_*N*_, *A*_*E*_, λ_*G*_, λ_*N*_, and σ_*E*_) needed to simulate the model are first optimized for healthy controls (Figure 6) using genetic algorithm (See Supplementary Material S2: Table 2 for Genetic Algorithm conditions). Once optimized for healthy controls, the parameters are then also used for the simulation of PD conditions (both OFF and ON). Furthermore the parameters (mentioned above) used to simulate the PD conditions are further optimized using a grid search algorithm (Supplementary Material S2: Table 3) to best fit the experimental behavior.

A repeated measures ANOVA was done to estimate statistical differences among subject groups (Controls, PD non-freezers and PD freezers) in different conditions (doorway and cues). Bonnferroni correction was applied to correct for type I error inflation. Additionally planned *t*-tests were conducted to measure statistical significance in specific cases. In simulations each subject group for a particular task condition was run for 50 trials and the averaged results are presented. All the simulations were done in MATLAB R2013a (Mathworks Inc.).

## Results

This section is organized as follows: We explain: (1) the effect of Utility and Risk observed in both the Cognitive and Motor Modules, (2) the effect of cognitive cues on gait and their contribution to step latency under conflicting situations such as while approaching the doorways, and finally (3) effect of cues as a source of cognitive load. These effects have been modeled in healthy controls, and PD patients under ON and OFF medication conditions.

### Value and risk as functions of the cues

**Figure 7:**
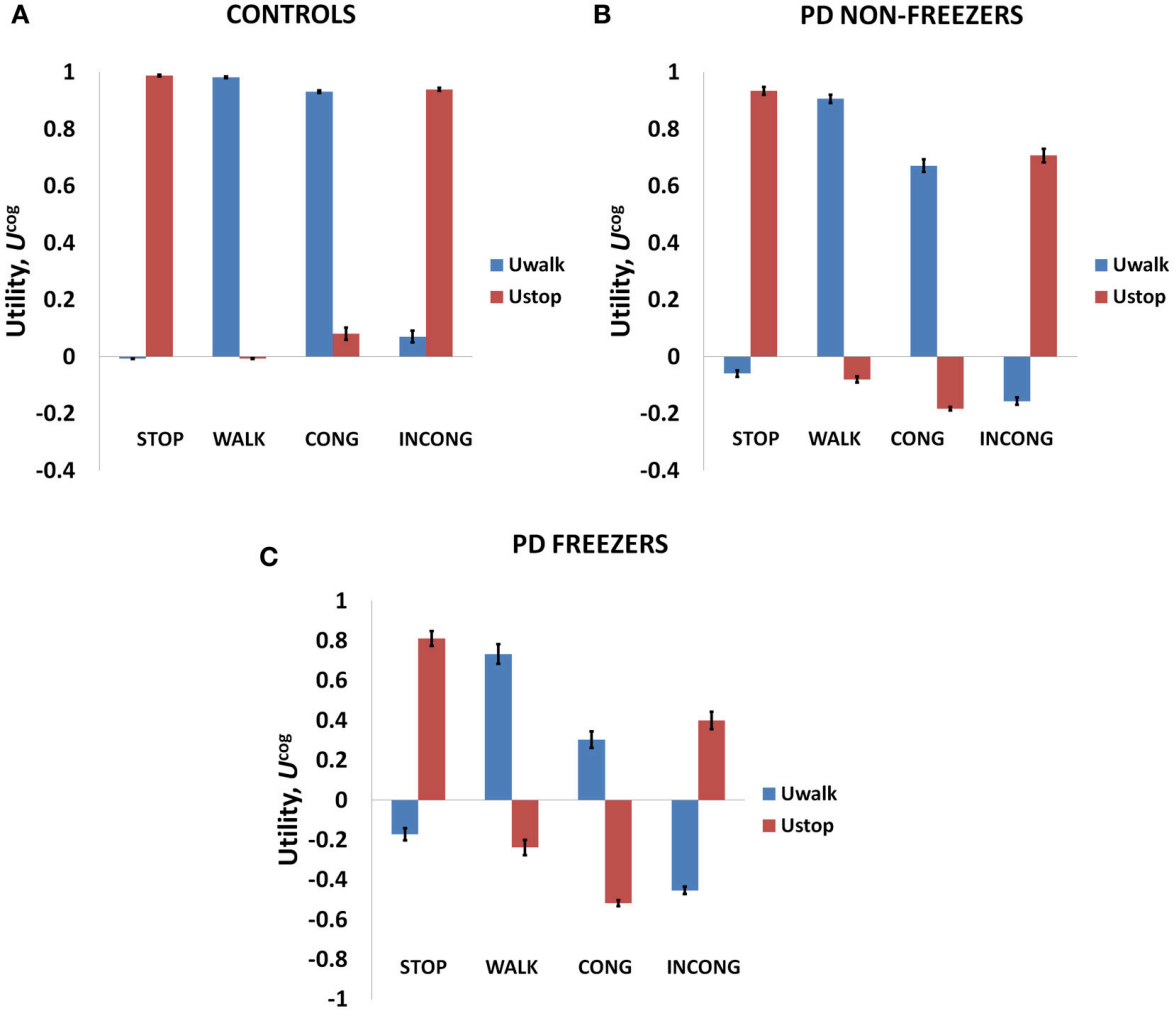
**The Utility obtained from the cognitive network for Controls (A)**, PD non-freezers **(B)** and PD freezers **(C)**. PD freezers exhibit lower U_*WALK*_ for the congruent cues compared to the simple cue WALK, reflecting uncertainty in their responses. Abbreviations: CONG, congruent cue; INCONG, incongruent.

As mentioned above, Matar et al. (2013) investigated the effect of the modified Stroop cues on gait latency. The subject groups tested were healthy controls, PD non-freezers and freezers. In our model simulations of Matar et al. (2013), the agent is trained to “walk” (as a response) for a congruent cue (stimulus) and “stop” for an incongruent cue. Utility associated with the cues represents the goodness associated with an action in presence of the said cue. We derive Value, Risk, and Utility measures associated with each of the cues. Analyzing the effects of simple and complex cues, we found the following: the association of a complex cue (congruent or incongruent) to the VR task can decrease the certainty associated with the action, as these associations are not pre-learnt. In Figures 7A–C, in PD freezers and non-freezers, it is evident that complex cues are associated with low Utility for both actions, walk [*F*_(8, 2)_ = 95.79, *p* < 0.05] and stop [*F*_(8, 2)_ = 97.53, *p* < 0.05)], suggesting increased uncertainty in their responses. Furthermore, the Utility is much lower in the freezers compared to the non-freezers (*t* = 5.04, *p* < 0.05).

**Figure 8:**
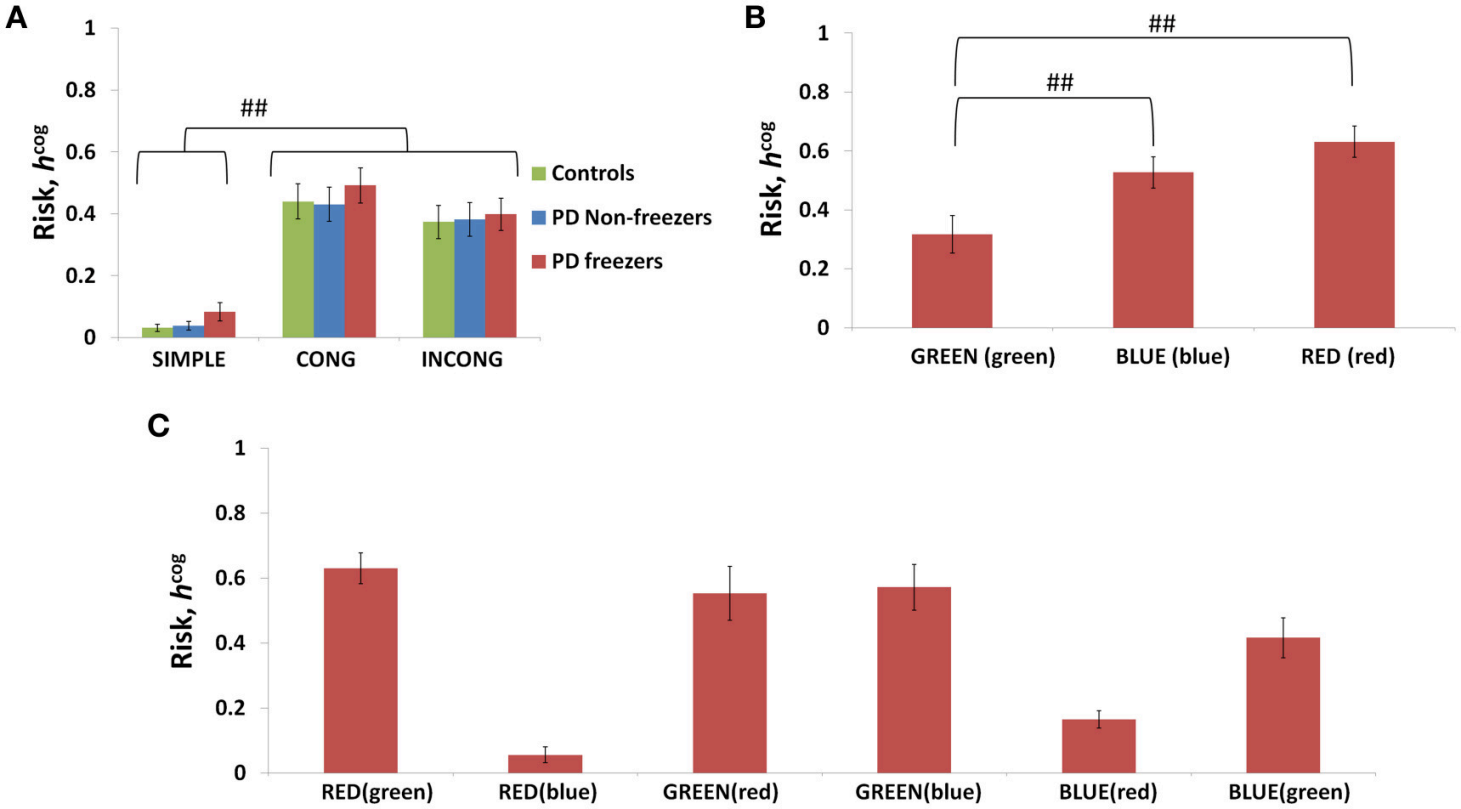
**Risk as function of cues (A)** presented shows higher Risks estimated in the case of the congruent and the incongruent cues in all the three subject groups. The Risk functions **(B,C)** associated with the congruent and incongruent cues respectively in PD freezers. The RED (red) shows the highest Risk compared to the other two congruent cues. Abbreviations: CONG, congruent cue; INCONG, incongruent. (^##^*p* < 0.05).

The conflict in the association between cues and actions determines the Risk and Utility magnitudes of a cue component. For example, the presentation of “RED (red)” makes the agent continue walking, but the red color is initially primed to the response stop. The weightage of influence provided by the ink color and word meaning, and their magnitude of conflict within these components for the different classes of cues (simple, congruent and incongruent) can be analyzed by their Risk magnitudes as seen in Figure 8A. The model estimates higher Risks for all three subject groups for congruent and the incongruent cues in comparison to the simple cues [*F*_(36, 2)_ = 21.74, *p* < 0.05]. The congruent cues have higher Risk which arises as a result of training the cue RED (red) to respond to “walk” whereas the inherent priming of the red stimulus (color or word) is to “stop.” This can be seen in Figure 8B which represents the Risk estimated by PD freezers for congruent cues in which RED (red) shows the highest Risk in comparison to BLUE (blue) and GREEN (green). The increased Risk observed in the model affects the Utility through Equation 14. So in order to show behavioral differences the risk sensitivity is modulated among the groups. PD freezers have been modeled to have higher risk sensitivity (α^*cog*^) in the cognitive loop (see Table 2).

Table 2**Parameter values for simulating the behavior seen in the Matar et al. (2013) and Shine et al. (2013d) experiments**.

**Table:** 

**Matar et al., 2013**					
	**Motor Loop**			**Cognitive Loop**	
	δ^*^	σ_*exp*_	α^*mot*^	δ^*^	α^*cog*^
Controls	–	0.5	0.5	–	0.1
PD Non-freezers	0.02	0.5	0.3	0.15	0.5
PD Freezers	**0.005**	**0.2**	**0.1**	**0.04**	**1**

**Table:** 

**Shine et al., 2013d**							
**Motor Loop**					**Cognitive Loop**		
**OFF**	δ^*^	σ_*exp*_	α^*mot*^	δ_*med*_	δ^*^	α^*cog*^	δ_*med*_
PD Non-freezers	0.02	0.5	0.3	–	0.15	1	–
PD Freezers	**0.003**	**0.1**	**0.1**	–	**0.08**	**7**	–
**ON**							
PD Non-freezers	0.02	0.5	0.3	0.001	0.15	1	0.001
PD Freezers	**0.003**	**0.1**	**0.1**	0.001	**0.08**	**1**	0.001

*The bold values highlight parameters differences in PD freezers in comparison to controls and PD non-freezers*.

### Utility and risk functions in relation to gait

**Figure 9:**
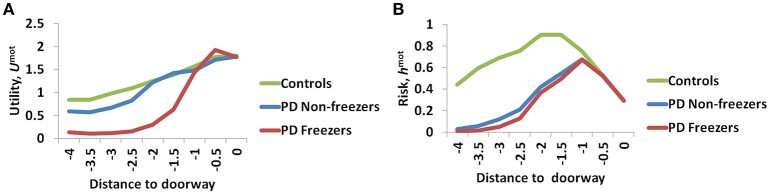
**The Utility (A)** and Risk **(B)** function for the Motor Module as a function of distance to the doorway. As seen in **(A)** the controls show the highest Utility along with a gradual change in the gradient, while PD freezers show almost no change in the Utility far away followed by a sharp decrease near the doorway. The Risk function **(B)** also peaks closer to the door in case of both the PD freezers and non-freezers.

The variation among controls, PD non-freezers and PD freezers is analyzed by examining the Utility (*U*^*mot*^) and the Risk (*h*^*mot*^) functions obtained from the Motor Module. The differences are presented as a function of the distance from the doorway in Figure 9.

The amount of uncertainty that the model estimates is computed for every subject group using Equation 18. The Utility measure is the highest for controls followed by PD non-freezers and then PD freezers (Figure 9A). While analyzing the subjective Risk measures from the model for different subject groups, and the corresponding α measure, we see that the subjective Risk computed is very high for healthy controls as seen in Figure 9B. The values of the sensitivity factor (α^*mot*^) are in Table 2. The temporal difference error (correlate of dopamine) is clamped in PD non-freezers and freezers, with the freezers having a stronger clamp than non-freezers. Additionally the Risk function seems to peak closer to the doorway for PD subjects, suggesting its role in controlling latency near the doorways.

### Effect of cognitive cues on motor activity

On extending the Cognitive Module's contribution to the Motor Module, the model predicts the conflict among the different cues, which can be estimated as the response (step latency) of the agent upon the presentation of a Stroop word.

#### Behavioral results

**Figure 10:**
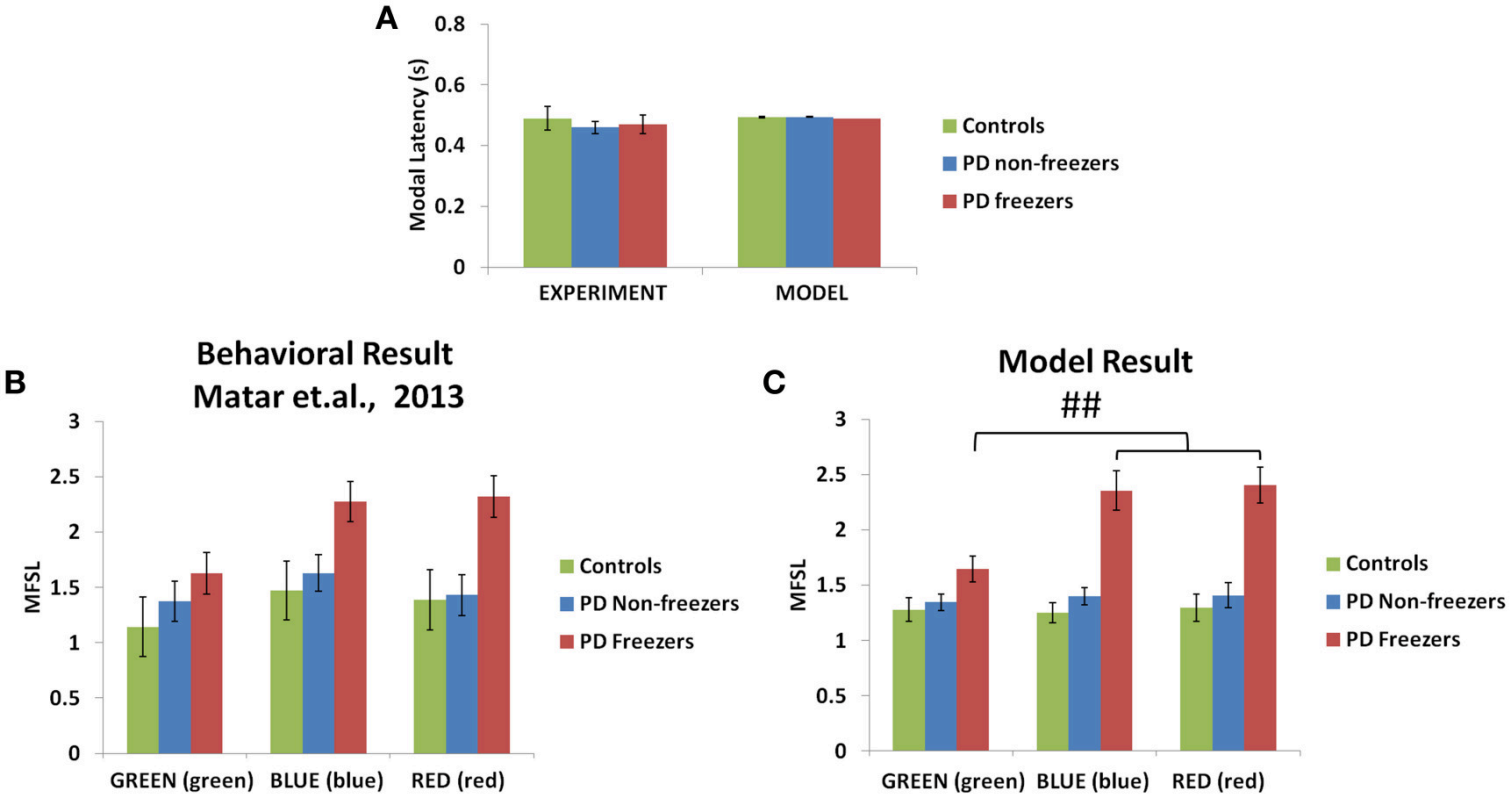
**Experimental (Matar et al., 2013) and Modeling data of modal latency (A)** observed in controls, PD non-freezers and PD freezers. The maximum scaled footstep latency (MFSL) exhibited on the presentation of the congruent cues as seen in the Matar et al. experiment **(B)** and the model **(C)**. It illustrates that PD freezers show increased latencies on the high conflict cues like RED (red) compared to the low conflict case GREEN (green). (^##^*p* < 0.05).

The model simulates the result of Matar et al. (2013) to understand the effect of cognitive cues on motor activity. Modal latency in Figure 10A shows no change in the latency among controls, PD non-freezers and PD freezers, similar to experimental results. This also augments the validity of the results obtained as the behavior in the model is not affected by the result of changes in the modal latency. The experimental results (Figure 10B) show that cues like GREEN (green) which have an implicit salience for “walk” response, evoke little or no change in the step latency for PD freezers. The RED (red) cue which has an implicit salience to “stop” response seems to increase the step latency of the PD freezers.

#### Model results

The model replicates this effect where the BLUE (blue) [*F*_(49, 2)_ = 1376.88, *p* < 0.05] and RED (red) [*F*_(49, 2)_ = 1048.01, *p* < 0.05] cues produced maximum footstep latency (MFSL) in the freezers in comparison to controls and non-freezers (Figure 10C). Consequently these are the two cues for which the Risk given by the Cognitive Module is high (Figure 8B). Several studies propose the presence of higher uncertain component of the environment as a reason for the inability to inhibit such latent behavior (Vandenbossche et al., 2011, 2012). From observing only the Cognitive Module, the uncertainty in the simulated PD condition arises in the model due to (a) training the Cognitive Network under clamped δ (dopamine) conditions (see δ^*^ in Table 2), and (b) by controlling the agent's sensitivity toward the Risk (*h*^*cog*^) associated with the cue using the parameter α^*cog*^. The risk sensitivity (α^*cog*^)in freezers is set higher than the other two groups, suggesting that the Risk taken into account for computing the Utility for a specific cue could be higher in freezers (Table 2). So, besides the Risk estimation being high, its accountability for behavior is also found to be high through our model.

### The influence of doorways on step latency

#### Behavioral results

**Figure 11:**
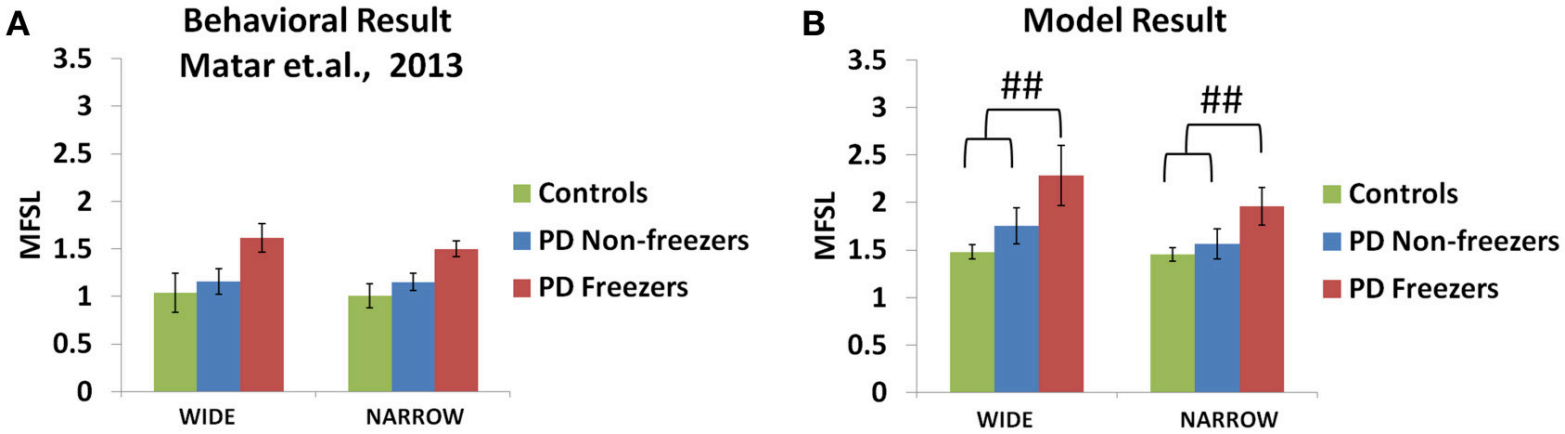
**Maximum footstep latency (MFSL) observed for both the narrow and the wide doorways in the experiments (A)** and the model **(B)**. The MFSL is higher for both the doorways for the PD freezers compared to the non-freezers and controls. (^##^*p* < 0.05).

The experiments reported in Matar et al. (2013) suggest that the PD freezers exhibit higher step latencies in both the wide and narrow doorway cases, with the narrow doorway being more significant than the wide doorway (Figure 11A) compared to controls and non-freezers. There seemed to be a doorway width and group interaction, which is enhanced in case of narrow doorways. Moreover, in the experiment the latency during navigating narrow doorways had good correlation to item 3 of FOG questionnaire score (FOG-Q3—“Do you feel as if your feet are glued to the floor while walking, making a turn or while trying to initiate walking”). There is no significant doorway- word cue interaction in the study suggesting that the cues might not be involved in affecting the doorway latency within the subject groups.

#### Model results

As mentioned previously in the *Methods* section all the cues presented to the agent appear in the region of 0–2 length units from the doorway. We study the effects of doorways on the step latency as the agent passes through them. The model captures the trends seen in the experiment in relation to the step latency exhibited by each subject group as they encountered a doorway of a specific type (Figure 11B). The wide [*F*_(49, 2)_ = 136.5, *p* < 0.05] and narrow [*F*_(49, 2)_ = 163.5, *p* < 0.05] doorways provoked increased step latencies in PD freezers compared to the controls and non-freezers. The behavioral performance in PD non-freezer and PD freezer conditions is simulated by using parameters described in Table 2. The temporal difference errors in both Cognitive and Motor Modules are clamped to represent PD conditions, although the level of clamping was different for the two modules (Table 2). In PD freezers the values of both the exploration factor (σ_*E*_) and the sensitivity of the motor Risk function (α^*mot*^) are lesser than in case of the controls and the non-freezers (Table 2), in contrast to the cognitive loop where the α^*cog*^ is higher for the PD freezers compared to the other subjects.

### Cognitive load and motor arrests

The effect of cognitive load on motor responses is a result of the ability of the subject to map cues to appropriate actions, depending on the nature of the cues. In this respect, simple cues are easily associated with their corresponding—walking or stopping. This is different for complex cues as mapping to actions is not straightforward.

#### Behavioral results

**Figure 12:**
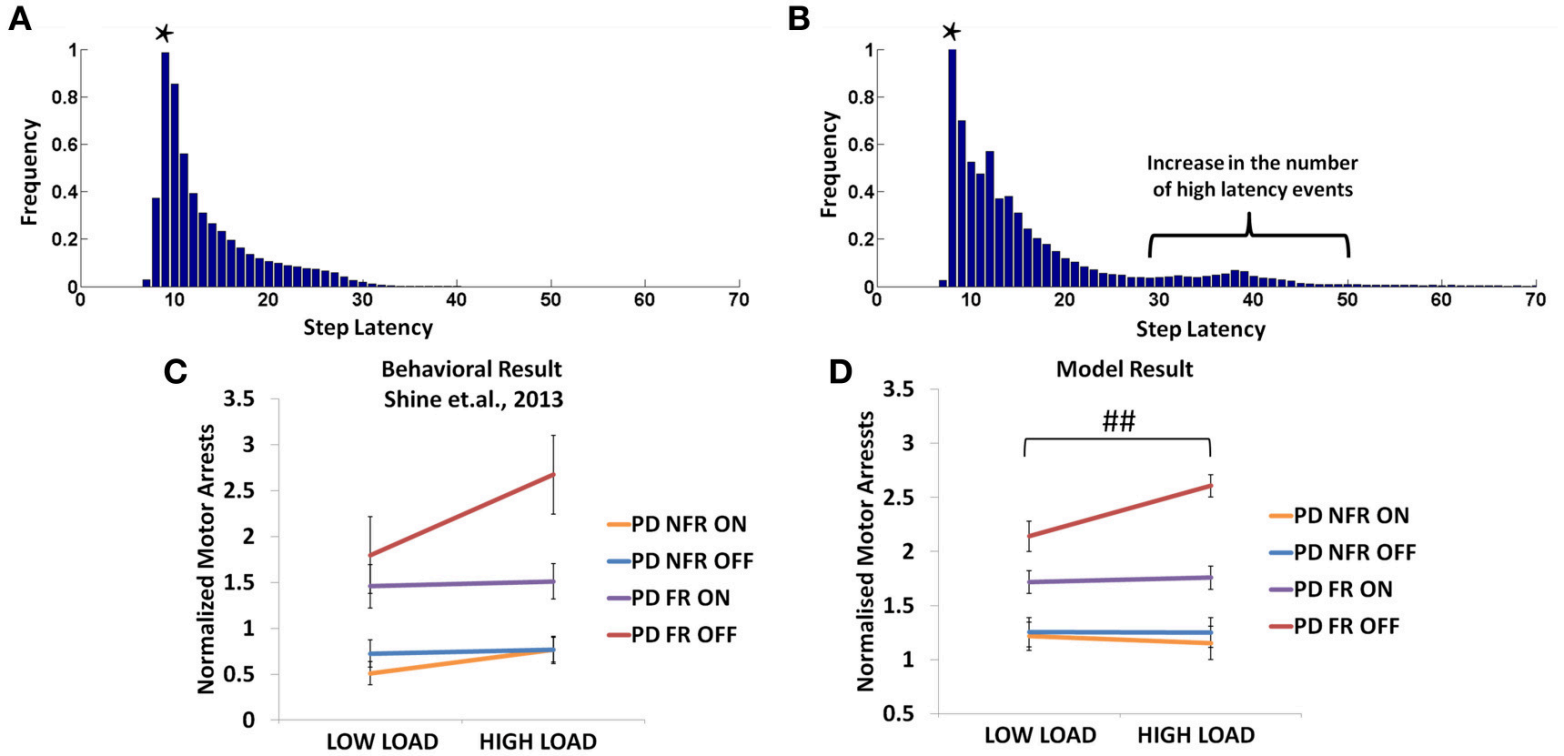
**The frequency distributions of step latency observed from the model for the PD non-freezers (A)** and the PD freezers where ^*^ represents the modal points **(B)**. Motor arrests seen in PD freezers and non-freezers under low and high levels of cognitive load in experiments **(C)** and the model **(D)**. The PD freezers (OFF) show a large number of motor arrests, which comes down under medicated conditions. PD non-freezers show no significant changes in the both the loads as well as the medication. (Abbreviation NFR, Non-freezer; FR, Freezer) (^##^*p* < 0.05).

In the Shine et al. (2013d) experiment, PD non-freezers and freezers were presented with cues both in the OFF and the ON medicated conditions. The trials were also counterbalanced among the patients such that a congruent cue is associated to “walk” and incongruent to “stop” and vice versa. According to the experiments, which were conducted on both PD freezers and non-freezers, the outcome of loading is evident from the number of motor arrests observed. The experiments were conducted with patients ON and OFF their dopamine medications. The PD freezers (OFF) showed the highest number of motor arrests (Figure 12C), with the tendency of freezing about 2.7 times more than the non-freezers. Though, PD freezers were generally more likely to suffer a motor arrest (both OFF and ON) compared to the non-freezers, the high load situation triggered more arrests in the PD freezers (Figure 12C). Similar to the previous experiment, there were also no significant differences in the modal latency between the PD freezers and the non-freezers.

#### Model results

In the model, a similar strategy is imposed and the trials including the low and the high load cues are extracted. The number of motor arrests is estimated using the distribution of the step latency (Figures 12A,B). As previously defined in the *Methods, a motor arrest is any event with step latency that is twice the modal (preferred) step latency of the subject*. The PD freezers seem to have higher frequency of higher step latency events especially in the regions of 30–50 in Figure 12B.

The modeling results are similar to experimental results (Figure 12D), where under high load scenario, the PD freezers OFF medication show maximum motor arrests, which is comparatively less in the low load case [*F*_(49, 1)_ = 4.30, *p* < 0.05]. Similar to the previously simulated experiment, the temporal difference errors (δ^*mot*^ and δ^*cog*^) in both the modules are clamped along with appropriate modulation of the sensitivities (α^*mot*^ and α^*cog*^) of the Risk function. The introduction of medication seems to bring down motor arrests in the freezers, suggesting that the DA medications play a role in lowering the number of such spontaneous events though its mechanism of action is still unknown. However, in the model, the addition of a medication factor (δ_*med*_) in the TD error eqn. (Equation 28) did not produce the same effects as the experiments. In addition to δ_*med*_factor, the sensitivity toward the uncertainty in the cues (α^*cog*^) had to be significantly reduced to simulate this behavior (Table 2). Such effects are not seen in the PD non-freezer case and the model predicts no significant changes in the motor arrests in both the medication as well as the load states.

**Figure 13:**
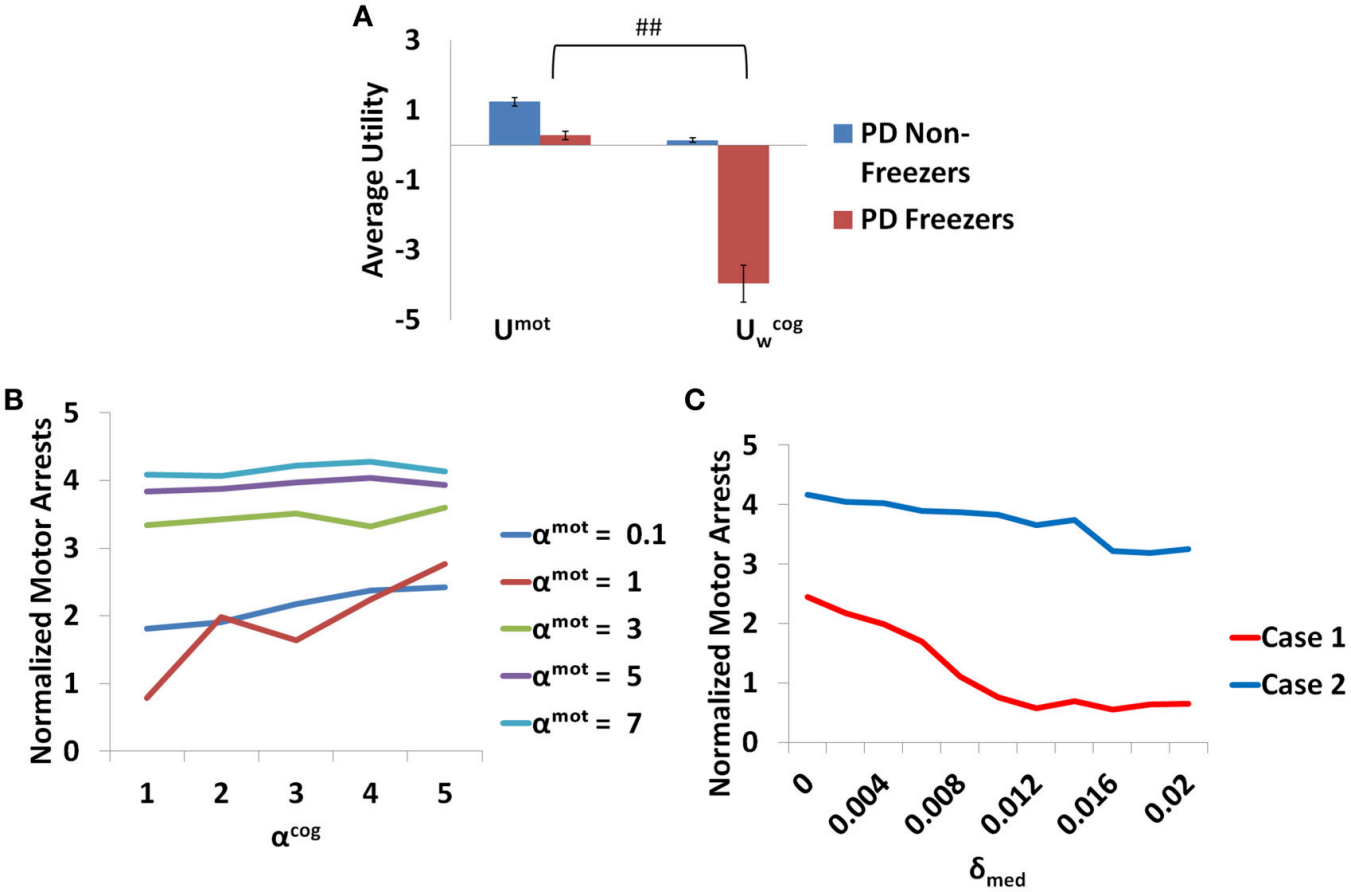
**The average Utility (A)** for both the PD non-freezers and the PD freezers during events of motor arrests triggered upon the presentation of a word cue. (^##^*p* < 0.05). The normalized motor arrests in the model **(B)** seen in PD freezers for different values of α^*mot*^ and α^*cog*^. **(C)** The trend for the motor arrest as a function of the medication factor (δ_*med*_) for two cases (Case1 α^*mot*^ = 0.1; α^*cog*^ = 7 and Case2 α^*mot*^ = 7; α^*cog*^ = 7).

Additionally the involvement of the Cognitive Module in motor arrests can also be ascertained by analyzing the Utility of the Cognitive and Motor Modules at the time of a motor arrest (Figure 13A). Since the Cognitive Module is active only during the presentation of words, instances where a motor arrest is elicited when a word cue is given are extracted and the contribution of the Motor and the Cognitive Module is visualized. It is clear that the average values of the Utility of walking ($U_{w}^{cog}$) of the Cognitive Module is much lower than Utility of the Motor Module (*U*^*mot*^) in case of PD freezers (*t* = 49.3, *p* < 0.05) compared to non-freezers. This further strengthens the claim that there is a shift to more cognition based decision during a freeze episode and understanding the role of these areas would lead to further insights into the phenomenon.

### The influence of risk sensitivities (α^*mot*^ and α^*cog*^) and medication (δ_*med*_) on motor arrests

The contribution of each parameter to the normalized motor arrests also reveals several trends (Figure 13B). Although lower α^*mot*^ values are used to simulate PD freezer conditions and are the optimal range for accounting for the experimental data, the trends suggest that higher α^*mot*^ would lead to a high number of motor arrests. The role of α^*cog*^ to elicit motor arrests seems to be more effective under conditions of low α^*mot*^ values where there is an increase in the number of motor arrests as α^*cog*^ increases. The model thus predicts an increase of α^*cog*^ to differentiate a control from a non-freezer but an increase in α^*mot*^ to increase the number of motor arrests. Medications are also found to play a role in decreasing the number of motor arrests, and in particular, a case of low α^*mot*^ is shown to better responds to medications (Figure 13C).

## Discussion

The current study simulates the impact of cognition on gait in healthy and PD subjects while navigating a virtual reality environment. In this task, an agent navigates a virtual corridor consisting of doorways, while simultaneously responding to word cues (Matar et al., 2013; Shine et al., 2013a,c,d). The word cues could be either simple (direct) or complex like Stroop words (color-word pair) mapped to an appropriate action (walk or stop). The classical Stroop task involves either naming the ink color of a color word or just reading the color out loud. There seems to be facilitation in the responses (indicated by shorter reaction times) when the word and its ink color match (a congruent case). In the case of an incongruent cue, which consists of a mismatch between the word and its color, there is an inhibition of response and increased reaction times (MacLeod, 1991). Conflict in this association arises due to the mismatch in association of action to WORD and COLOR aspects of complex cues. Thus, our study attempts to model the effect these interactions on gait execution. The model uses an actor-critic based reinforcement learning (RL) model which performs Utility-based decision making. An extensive literature supports the function of the BG system as a reinforcement learning engine (Albin et al., 1989; Frank et al., 2004; Chakravarthy et al., 2010; Chakravarthy and Balasubramani, 2014).

The main observations from the model are as follows

1. Modal latency is the same among all the subject groups and match experimental results. This stresses the fact that the results obtained from the model are a consequence of changes due to the introduction of task parameters (cues and doorways) and not due to inherent differences among the subject groups. Freezing is a paroxysmal event such that observed motor arrests come as a result of a shift to higher latencies. This can be considered as the involvement of higher cognitive areas over the action which in the model can come from the intervention of the cognitive module (Vandenbossche et al., 2012). This is also evident from Figure 13A where the Utility for the cognitive network during motor arrests in PD freezers is very low compared to the non-freezers suggesting higher involvement of cognition in an event of freezing.
2. The Utility associated with a cue in the Cognitive Module provides a measure of cue saliency in the model. This measure then affects reaction times (MFSL) elicited by PD freezers, which is increased under situations of high conflict [RED (red)]. The factors contributing to a very low Utility for a cue is predicted to cause a freezing event with a high probability. In general PD subjects present a cognitive control deficit especially while monitoring conflict in the task (Bonnin et al., 2010). Studies show there are certain situations that can be resource demanding, either attentional resources (Brown and Marsden, 1988; Woodward et al., 2002) or neuromodulator resources which can lead to a breakdown in the processing ability of the subjects.
3. Similarly, in the case of Motor Module, the doorways induce more freezing behavior in PD freezers in comparison with controls and non-freezers. Doorways by themselves have been a factor for eliciting changes in gait activity in PD freezers especially parameters like stride/step length that tends to be lower in the freezer group.
4. Motor arrests seem to be higher in PD freezers compared to non-freezers and increase significantly under conditions of high loads (trials with complex cues). Additionally the behavioral data from both the experiments are captured by the same set of parameters.

### Utility codes conflict

We use a Utility-based decision making model to analyze the effects of cognitive load on gait. The sole output of the proposed Utility-based decision making system is step latency. Step latency is selected to maximize Utility and minimize Risk. The Utility-based decision making approach has been previously shown to explain PD motor impairment in precision grip and freezing of gait (Gupta et al., 2013; Muralidharan et al., 2013). As seen from Equation (7), Utility in the model is a weighted combination of Value and Risk functions. Value (Q^*^) is the expectation of rewards associated with the state (word/view), and Risk captures the uncertainty associated with the state and rewards. Risk in the Cognitive Module is modeled to measure the uncertainty associated with the state, indirectly from the probability of selecting an action (*p*_*w*_ and *p*_*s*_) obtained from the Value function (*Q*^*cog*^). Here, the state uncertainty comes due to the Stroop task where the mapping of the color and word to the actions walk and stop can be conflicting for several input stimuli. In the Motor Module the Risk function captures the action uncertainty as a function of the view vector (ϕ). The action uncertainty is often a result of the tradeoff between accuracy and speed, in this case manifest as high step latency close to the doorway. So the reason for the higher Utility estimated in the model for healthy controls could be that their Value computation is higher, or contribution of Risk to Utility computation could be lower. Risk computed here is very specific to patient groups as well as sensory states (word vs. view). This is seen in the Figure 9B where Risk function represents the environmental context in the healthy controls effectively, but becomes sub-optimal when simulated under PD condition with clamped dopamine signal. Also note that this Risk function can replicate experimental data only when scaled by the optimal α values (Table 2). Hence the effective Risk function is the product of the risk sensitivity (α^*mot*^/α^*cog*^) and the Risk measure itself.

In the case of Cognitive Module, uncertainty is especially high for complex cues. In Figure 8B, the congruent cues seem to have high Risk and specifically RED (red) has the highest. The association of the RED (red) to the action walk as set in the described experimental task contradicts the implicit heuristic of associating the color red to the action “stop.” In the model simulations with the incongruent cues, BLUE (red) and RED (blue) seem to have a low measure of Risk (Figure 8C). The experimental setup associates the incongruent cues to action stop. The facilitation of the red word and color to the action stop when presented as a part of an incongruent cue, leads to a low Risk. In the case of blue, we propose that its neutrality may not contradict the mapping to the associated action as strong as red, and hence develops a low Risk measure. Thus, Utility forms a strong indicator of conflict and its proper estimation is necessary for optimal behavior.

### Model predictions

#### Role of the cognitive module in motor arrests

It is evident from Figure 13 that there is an increase in the contribution of the Cognitive Module to action selection in comparison to the Motor Module. The model predicts that PD freezers tend to rely more on the cognitive areas to perform the task. It is known that there is a shift toward more cortical resources during the situations that give rise to a freeze episode. Brain imaging studies have shown the recruitment of the posterior parietal cortex, dlPFC, and vlPFC (dorsolateral and ventrolateral prefrontal cortex), the anterior insula and the dorsal cingulate while performing the virtual reality task (Shine et al., 2013b). The role of anterior cingulate in resolving conflict has been well studied and especially thought to mediate process selection in the Stroop task (Pardo et al., 1990). Interestingly the anterior insular cortex has been investigated as a potential source for conscious error monitoring (Preuschoff et al., 2008; Ullsperger et al., 2010). This brain region was found to be implicated in generating autonomic responses in relation to balancing effortful tasks. Since the Utility in our model helps resolve the level of conflict in the task, we presume that these areas must be overcompensating for estimating the correct responses and thus preventing the subjects, especially the PD freezers, from proper gait execution.

A close analysis of the pattern of dopamine loss in PD patients indicates a relatively higher dopamine loss in putamen which controls the motor loop, compared to the caudate which controls the cognitive loop (Kish et al., 1988). This distinction is reflected in model parameters δ^*mot^ and δ^*cog^, which denote maximum permissible dopamine levels in motor and cognitive loops respectively. Note that δ^*mot*^ values are smaller compared to the δ^*cog*^ values in both the PD non-freezers and freezers (Table 2). This pattern of loss could be exaggerated in PD freezers especially in the motor areas, leading to increased postural defects, increased incidence of falling and gait variability (Bloem et al., 2004; Jacobs et al., 2009). Medication seems to be another factor whose effect on freezing behavior seems to be difficult to comprehend. There have been instances in PD freezers where dopamine medications have alleviated the symptoms, though in some cases it had no effect, and in others the symptoms worsened like in ON state freezing (Nonnekes et al., 2015). This is an important aspect of freezing pathology that has to be understood in order to improve therapeutic strategies. The model predicts that there could be conditions where medications could be more effective than in other cases (Figure 13C). It is possible from the two cases seen in the figure (Case 1: α^*mot*^ = 0.1; α^*cog*^ = 7 and Case 2: α^*mot*^ = 7; α^*cog*^ = 7) that we can categorize the patients based on theses parameters, i.e., dopamine responsive regime (Case 1) and dopamine insensitive regime (Case 2). These parameters reflecting the risk sensitivity of the patients need to be deeply investigated for their neurobiological correlates. The significance of Risk Sensitivity parameters in the model is elucidated further below.

#### Risk sensitivity parameters (α^*mot*^ and α^*cog*^)

The parameters α^*mot*^ and α^*cog*^ which correspond to risk sensitivity in each module seem to bring about the behavioral differences between controls, non-freezers and freezers. These Risk measures capture the variability in rewards (high when associating a cognitive or motor cue with a corresponding response) sampled through time due to the agent's response-execution strategy. On analyzing the cognitive and motor loops, the following trends for α emerge. The healthy controls are more risk averse than PD patients with respect to the Motor Module (Table 2). Contrastingly, the PD patients are highly risk aversive with respect to the Cognitive Module.

Our earlier modeling studies on motor functions of PD (Gupta et al., 2013; Muralidharan et al., 2013) show a lower magnitude of the risk sensitivity parameter in PD patients. Modeling efforts suggest that risk sensitivity correlates with the levels of serotonin in the motor areas (Balasubramani et al., 2014, 2015). Incidentally the concentration of serotonin and its derivatives in PD have been shown to be lower in the cerebro-spinal fluid, with a strong correlation with freezing of gait (Tohgi et al., 1993). But, we need to tease apart risk sensitivity measures for the motor and cognitive loops individually. We see reduced risk sensitivity measure in the motor loop, accounting for the reduced risk aversive and increased risk seeking nature of the PD patients. In contrast, we see increased risk aversiveness in the Cognitive Module. If the previous hypothesis of reduced serotonin levels to reduced risk aversiveness in the motor areas is generalized, the cognitive areas should also have reduced serotonin levels. But as our model predicts increased risk aversiveness in cognition, the following are some possibilities of their pathophysiology.

It could be that there are differential changes in serotonin levels in the cognitive and motor areas, the former containing higher serotonin levels associated with risk aversive behavior, and the latter containing reduced serotonin levels. There have been reports showing differential loss of the expression of certain serotonin markers in the caudate and the putamen in PD (Kish et al., 2008). The caudate could be considered to be part of the cognitive loop due to its projections from frontal areas and putamen part of the motor loop as it receives projections from the motor cortex (Parent and Hazrati, 1995). Hence increased risk aversiveness reported in the study for the cognitive loop relates to increased risk sensitivity measure in these areas. This might give rise to an altered paradigm of decision making in the cognitive loop where subjects take more Risk into account upon the introduction of other tasks while walking and maintaining posture. On the other hand this could also force subjects to adopt a posture–second strategy where the importance given to postural maintenance is less compared to cognitive ability suggesting increased risk-seeking behavior in PD patients in the motor side (Bloem et al., 2006). Therefore, besides dopamine, this model suggests the need to conduct experiments to measure serotonin levels within the Cognitive and Motor Modules, and how they relate to the risk sensitivity. Many studies relate different kinds of uncertainty to the effects of neuromodulators such as acetylcholine (Ach) to the expected uncertainty, norepinephrine (NE) to the unexpected uncertainty both in the cortex and basal ganglia (Yu and Dayan, 2005), serotonin to modulate expected uncertainty in the BG (Balasubramani et al., 2014, 2015). It is plausible that these neuromodulators have differential action on PD gait and therefore merit a close and comprehensive study.

#### Model limitations and future directions

Although the model replicates the behavior of controls and PD subjects under this paradigm, several additional neural level details as listed below can be potentially included. The model does not have an explicit representation of the cortex as it only includes the representation of cortical inputs for both the networks in the form of a word vector for the cognitive loop and the view vector for the motor loop. The Cognitive Module might involve the dorsolateral prefrontal cortex (DLPFC), the posterior parietal cortex and the caudate of the BG, while the Motor Module might include the motor cortex (M1), premotor area (PMA), and the putamen of the BG (Shine et al., 2013b). The sub-cortex receiving inputs from the cortex controls the gait centers in the brainstem through the output nucleus globus pallidus interna (GPi). GPi in turn controls the downstream brain stem regions (Mesencephalic locomotor regions) responsible for rhythm generation and maintenance of gait (Shine et al., 2013b). The model compares results based on the behavior to the VR tasks which only simulate the effect of locomotion. Although there seems to be a good correlation between the VR tasks and the timed up and go tasks in PD freezers (Shine et al., 2013a), especially the duration of motor arrests, it is necessary to quantify the model for actual walking tasks. Furthermore, in modeling perspective we could introduce a downstream gait model to understand changes in the dynamics of locomotion in PD patients.

Furthermore, the basal ganglia module in our model is an abstract version that could be developed to a more detailed neural network model (Balasubramani et al., 2015) elaborating the role of the different nuclei in eliciting freezing behavior. Additionally the interaction between the different cortical loops in our model occurs only at the level of the output of the basal ganglia (at the GPi/thalamic level), though interactions at cortex, striatum have also been reported to encompass cortico-striatal convergence (Guthrie et al., 2013). A network model including these areas would provide a better understanding of PD gait. It may also suggest neural targets for drug delivery in a patient-specific manner.

## Author contributions

VM, Conceiving, developing the model, data analysis and manuscript preparation. PB, Conceiving, developing the model, data analysis and manuscript preparation. VC, Conceiving, developing the model, data analysis and manuscript preparation. MG, reference data and manuscript preparation. SL, reference data and manuscript preparation. AM, reference data and manuscript preparation.

### Conflict of interest statement

The authors declare that the research was conducted in the absence of any commercial or financial relationships that could be construed as a potential conflict of interest.
