# Supplementary material for: A Neurocomputational Model of the Effect of Cognitive Load on Freezing of Gait in Parkinson's Disease
Source: Front Hum Neurosci. 2017 Jan 9;10:649. doi: 10.3389/fnhum.2016.00649 (PMC5220109; doi:10.3389/fnhum.2016.00649)
Supplement: Supplementary file 1 [file Table1.DOCX]

Supplementary Material S2

**A NEUROCOMPUTATIONAL MODEL OF THE EFFECT OF COGNITIVE LOAD ON FREEZING OF GAIT IN PARKINSON’S DISEASE**

Vignesh Muralidharan, Pragathi Priyadharsini. B, V. Srinivasa Chakravarthy^*^, Simon J. G. Lewis, Ahmed A. Moustafa

*** Correspondence:** V. Srinivasa Chakravarthy: schakra@iitm.ac.in

Supplementary Data

**Appendix B**

**Table 1** List of parameters for simulating the Cognitive and the Motor Modules for both experiments

| Cognitive Loop | Parameter values | Motor Loop | Parameter values |
| --- | --- | --- | --- |
| *A*_Q_^cog^ | 1 | *A*_Q_^mot^ | 5 |
| *λ*^cog^ | 3 | *A*_h_^mot^ | 5 |
| *η*^cog^ | 0.1 | *λ*^mot^ | 0.05 |
| *a*_p_ | 0.25 | *η*^mot^ | 0.1 |
|  |  | *A*_G_ | 1 |
|  |  | *A*_N_ | 1 |
|  |  | *A*_E_ | 1 |
|  |  | *λ*^vel^ | 3 |

The Genetic Algorithm([Goldberg 1989](#_ENREF_17)) option set for optimization is given in the following table. Optimization toolbox 6.0, Matlab R2013a, The Mathworks Inc. is used.

**Table 2** Options set for the GA tool

| Option | value | | | | | | | | | | | |
| --- | --- | --- | --- | --- | --- | --- | --- | --- | --- | --- | --- | --- |
| Population Size | 20 | | | | | | | | | | | |
| Crossover fraction | 0.8 | | | | | | | | | | | |
| Elite count | 4 | | | | | | | | | | | |
| Generation time | 100 | | | | | | | | | | | |
| Function tolerance | 1 e-6 | | | | | | | | | | | |
| Bounds |  | *A*_Q/h_^mot/cog^ | *A*_G_ | *A*_N_ | *A*_E_ | _,_*λ*_G_ | *λ*_N_ | *λ*_Q/h_^mot/cog^ | *α*^mot^ | *α*^cog^ | *γ* | *σ* |
|  | Upper | 0 | 0 | 0 | 0 | 0 | 0 | 0 | 0 | 0 | 0 | 0 |
|  | Lower | 10 | 10 | 10 | 10 | 1 | -1 | 1 | 1 | 1 | 1 | 1 |
| Cost function | (Expt measure - Sims measure)^2^ $C=\sum_{i=1}^{5} (\mathrm{ex}_{i}-sims_{i})^{2}$   *'ex'*  here refers to the mean experimental maximum foot step latency (MFSL) values for doorways and the cues (1 – wide, 2 – narrow, 3 – GREEN (green), 4 – BLUE (blue), 5 – RED (red)) and *'sims'* is the model’s output to a set of parameter values | | | | | | | | | | | |

**Table 3** The Grid search parameters and ranges for the optimizing PD Non-freezers and PD freezers (both ON and OFF)

| Cognitive Loop | Lower | Upper |
| --- | --- | --- |
| *δ*^cog^ | 0 | 1 |
| *α*^cog^ | 0 | 7 |
|  |  |  |
| Motor Loop |  |  |
| *δ*^mot^ | 0 | 1 |
| *α*^mot^ | 0 | 7 |
| *σ* | 0 | 1 |
|  |  |  |
| Other |  |  |
| *δ*_med_ | 0 | 0.02 |
