# Supplementary material for: A Neurocomputational Model of the Effect of Cognitive Load on Freezing of Gait in Parkinson's Disease
Source: Front Hum Neurosci. 2017 Jan 9;10:649. doi: 10.3389/fnhum.2016.00649 (PMC5220109; doi:10.3389/fnhum.2016.00649)
Supplement: Supplementary file 2 [file DataSheet1.DOCX]

Supplementary Material S1

**A NEUROCOMPUTATIONAL MODEL OF THE EFFECT OF COGNITIVE LOAD ON FREEZING OF GAIT IN PARKINSON’S DISEASE**

Vignesh Muralidharan, Pragathi Priyadharsini. B, V. Srinivasa Chakravarthy^*^, Simon J. G. Lewis, Ahmed A. Moustafa

*** Correspondence:** V. Srinivasa Chakravarthy: schakra@iitm.ac.in

Supplementary Data

The heading vectors (*H_i_^sec^*) for each visual sector in both the fields of vision are given with respect to the current orientation ($H_{curr}=\frac{\Delta Z(t)}{\left\|\Delta Z(t)\right\|}$) by the following expression.

| - $H_{l}^{sec}=\text{ R}_{l}H_{curr}$ | (1) |
| --- | --- |

where *R*_l_ is a rotation matrix which is given as $\left(\begin{matrix} \cos\Theta_{l} & \sin\Theta_{l} \\ -\sin\Theta_{l} & \cos\Theta_{l} \end{matrix}\right)$ and *Θ*_l_ represents the angle made by each of the 50 sectors with the current heading direction (*H*_curr_). The slope of each of these visual vectors is calculated using eqn A.2.

| - $m_{i}=\frac{\Delta Y_{l}}{\Delta X_{l}}$ | (2) |
| --- | --- |

Using the information from the position of the center of the doorway (*x*^door^ *,y*^door^), its width (*w*^door^) and the slope of the orientations vector, it is possible to estimate whether a given visual vector hits the wall or the doorway.

| - $H_{l}^{door}=\frac{Y^{door}-Y}{m_{i}}+X$ | (3) |
| --- | --- |

Here (*X, Y*) represent the current position of the agent and *Y*^door^ corresponds to the Y coordinate of the center of a doorway. The view vector is then given as

| -  | (4) |
| --- | --- |
